# Predictive Molecular Design and Structure–Property Validation of Novel Terpene-Based, Sustainably Sourced Bacterial Biofilm-Resistant Materials

**DOI:** 10.1021/acs.biomac.2c00721

**Published:** 2023-01-04

**Authors:** Valentina Cuzzucoli Crucitti, Aleksandar Ilchev, Jonathan C. Moore, Harriet R. Fowler, Jean-Frédéric Dubern, Olutoba Sanni, Xuan Xue, Bethany K. Husband, Adam A. Dundas, Sean Smith, Joni L. Wildman, Vincenzo Taresco, Paul Williams, Morgan R. Alexander, Steven M. Howdle, Ricky D. Wildman, Robert A. Stockman, Derek J. Irvine

**Affiliations:** † Centre of Additive Manufacturing, Department of Chemical and Environmental Engineering, University of Nottingham, University Park, NottinghamNG7 2RD, U.K.; ‡ School of Chemistry, University of Nottingham, University Park, NottinghamNG7 2RD, U.K.; § Advanced Materials and Healthcare Technologies, School of Pharmacy, University of Nottingham, University Park, NottinghamNG7 2RD, U.K.; ∥ National Biofilms Innovation Centre, Biodiscovery Institute and School of Life Sciences, University of Nottingham, University Park, NottinghamNG7 2RD, U.K.

## Abstract

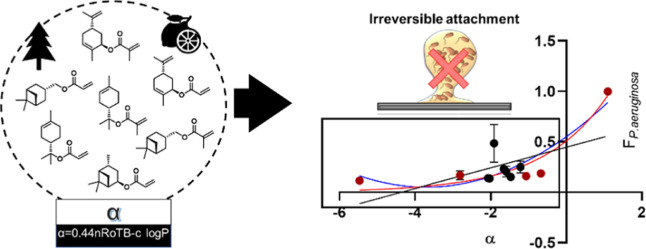

Presented in this work is the use of a molecular descriptor, termed the α parameter, to aid in the design of a series of novel, terpene-based, and sustainable polymers that were resistant to biofilm formation by the model bacterial pathogen *Pseudomonas aeruginosa*. To achieve this, the potential of a range of recently reported, terpene-derived monomers to deliver biofilm resistance when polymerized was both predicted and ranked by the application of the α parameter to key features in their molecular structures. These monomers were derived from commercially available terpenes (*i.e.*, α-pinene, β-pinene, and carvone), and the prediction of the biofilm resistance properties of the resultant novel (meth)acrylate polymers was confirmed using a combination of high-throughput polymerization screening (in a microarray format) and *in vitro* testing. Furthermore, monomers, which both exhibited the highest predicted biofilm anti-biofilm behavior and required less than two synthetic stages to be generated, were scaled-up and successfully printed using an inkjet “valve-based” 3D printer. Also, these materials were used to produce polymeric surfactants that were successfully used in microfluidic processing to create microparticles that possessed bio-instructive surfaces. As part of the up-scaling process, a novel rearrangement was observed in a proposed single-step synthesis of α-terpinyl methacrylate *via* methacryloxylation, which resulted in isolation of an isobornyl–bornyl methacrylate monomer mixture, and the resultant copolymer was also shown to be bacterial attachment-resistant. As there has been great interest in the current literature upon the adoption of these novel terpene-based polymers as green replacements for petrochemical-derived plastics, these observations have significant potential to produce new bio-resistant coatings, packaging materials, fibers, medical devices, etc.

## 1 Introduction

In the recent years, polymers obtained from renewable resources have attracted much attention as the search to provide sustainable alternatives to petrochemical-derived polymers has intensified.^1−3^ This surging interest in sustainable polymers has been stimulated by the potential for depletion of fossil fuel stocks, the growing concern over persistent plastic pollution in the environment, and climate change.^4^ However, despite this growing interest in bioderived and renewable plastics, sustainable polymers still constitute less than 10% of the total volume of commercial polymer production.^1,3^ Meanwhile, recent predictions have defined that crude oil and gas feedstocks are expected to be entirely depleted within the next century. This highlights the need for commercial polymer producers to find new, renewable feedstocks.^3^ This will involve the conversion of renewable, preferably non-food competing feedstocks into novel monomers and/or polymers, either directly or after some chemical manipulation. The aim would be that these will give a range of novel macromolecular structures that can both (a) serve as drop in replacements for current high-volume plastics and (b) exhibit new and useful properties currently unobtainable from today’s petrochemical-based materials.^5^

To date, terpene-based monomers have played a key role in the construction of such sustainable functional polymers that exhibit a wide range of desirable mechanical and thermal properties.^6−8^ This variety in material properties has been attributed to either their structural diversity and/or the presence of alternative chemical functionalities, when compared to the standard petrochemical derivatives used to make most current common polymers. Consequently, they have been used in the synthesis of block and random copolymers with a broad range of properties,^9,10^ novel polycarbonate materials,^11^ and new biodegradable/sustainable surfactants.^12^

Another interesting aspect associated with this class of molecules is that they are considered to have intrinsic bio-instructive properties, such as anti-inflammatory, antiviral, antioxidant, and antibacterial characteristics.^13−19^ For instance, many examples of antimicrobial formulations based on these materials have been reported in the literature.^20−25^ This is of particular interest due to the increasing challenge for global healthcare systems which is attributable to the build-up of antimicrobial resistance. The subsequent absence of access to effective antimicrobial agents to support medical treatments is of sufficient worldwide concern. The Organization for Economic Co-operation and Development (OECD) report (Stemming the Superbug Tide, 7 Nov 2018) has predicted that 2.4 million people in Europe, North America, and Australia will die from infections with resistant microorganisms in the next 30 years.^26−28^ Moreover, a review focusing on the potential economic impact of antimicrobial resistance has forecast that if unchecked, antimicrobial resistance could cost the global economy up to US$3.5 billion per year.^27^

Chronic infections caused by antibiotic-resistant pathogen biofilm formation on implanted medical devices are especially refractive to eradication. On reaching a surface, bacterial cells transition through several stages from a free-living “planktonic lifestyle” to a surface-attached biofilm community where the cells are embedded in a self-generated extracellular matrix. Biofilm development begins with reversible attachment of cells and progresses *via* irreversible attachment to microcolony growth, biofilm maturation, and dispersal.^29,30^ Biofilms enable bacteria to evade phagocytosis, oxidative stresses, nutrient/oxygen restriction, and concentrate nutrients; engage in interspecies competition; and exhibit high-level tolerance toward antimicrobial agents.^31,32^

To date, many of the strategies used to prevent biofilm build-up and reduce the potential for adverse medical consequences have involved the use of biocidal additives such as silver ions.^32−34^ In contrast, work by Alexander’s group has led to the discovery of a class of (meth)acrylate polymers with broad resistance to bacterial biofilm formation which is not bactericidal or bacteriostatic, i.e., does not kill bacteria or inhibit their growth. For a multi-antibiotic-resistant pathogen such as *Pseudomonas aeruginosa*, these biofilm-resistant (meth)acrylates inhibit the transition from reversible attachment and so prevent biofilm formation.^35^ These were identified by first generating large combinatorial polymer (homo- and co-polymer) microarrays from commercially available (meth)acrylate and acrylamide monomers. These were then used to conduct high-throughput biofilm assays, and they identified a significant number of “hit” monomer types (*i.e.*, biofilm-resistant). Subsequent review of molecular structural descriptors of the “hits”, either by direct extrapolation or application of machine learning algorithms, identified key characteristics that related to the delivery of the desired bio-performance of the monomer and hence the polymer structure.^36,37^ These original assays included an investigation of biofilm formation by different bacterial pathogens. This study demonstrated that there was no relationship between the biofilm resistance and water contact angle (WCA), as the “hit” polymers were all typically in the 80–90° WCA range. Rather, a relationship which was referred to as the α parameter (eq 1) was noted to strongly correlate with resistance biofilm development observed from these monomer candidates for both Gram-negative (*P.seudomonas aeruginosa*, *Proteus mirabilis,* and *Escherichia coli*) and Gram-positive pathogens. The α parameter relationship was composed by the calculated logarithm of the partition coefficient (clog *P*) and number of rotatable bonds (*nR*_0_*TB*).
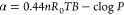
1

This relationship contains parameters relating to the hydrophobicity (clog *P*) of the material and its molecular flexibility (number of rotatable bonds = *nR*_0_*TB*).

The work reported in the present study extends this initial review of commercially available, petrochemically derived monomers to design and validate the biofilm resistance of a palette of monomers derived from renewable and sustainable resources (terpenoids). This involved the application of the α parameter to molecule types not within the original data set to screen the potential structural types, to predict which of these are likely to exhibit a high level of resistance to biofilm formation. These were then synthesized and ranked according to their α parameter, using eq 1, and their performance was validated by *in vitro* testing against *P. aeruginosa* as the model pathogen. The newly synthesized monomers have been derived from commercially available terpenes, including α-pinene, β-pinene, and carvone.

## 2 Materials and Methods

### 2.1 Materials

1,6-Hexanediol diacrylate (HMDA) (80%), polyvinyl alcohol (25 kDa), and 2,2 dimethoxy-2-phenylacetophenone (photoinitiator) and poly(hydroxyethyl) methacrylate were purchased from Sigma-Aldrich. Ethyl acetate (EA), isopropyl alcohol (IPA), and dichloromethane (DCM) were obtained from Fisher Scientific. All chemicals were used as received without further purification. The synthetic procedures and full characterization data for monomers A–G have been reported previously by the authors.^38,39^

### 2.2 Synthetic Procedures

#### 2.2.1 Synthetic Procedures to Produce Target Monomers

The general workflow for preparation of terpene monomers was as follows: (a) synthesis of an (iso)bornyl–bornyl methacrylate mixture *via* methacryloxylation, which was the proposed single-step route to this target product that was found to give rise to a mixture of monomers, (b) the isolation of the target monomer *via* esterification of this monomer from terpenes, a method known to produce high yields on two different scales of our process, and (c) the synthesis of bornyl methacrylate to confirm both spectroscopic assignments and biological performance.

### 2.3 Synthesis of the Terpene Monomers

#### 2.3.1 Proposed Single-Step Synthesis of α-Terpinyl Methacrylate *via* Methacryloxylation Which Resulted in Isolation of an Isobornyl–Bornyl Methacrylate Mixture

2,6-Di-*tert*-butyl-4-methylphenol (110 mg, 0.50 mmol, 0.005 equivalents) was dissolved in DCM (250 mL) with stirring. (−)-α-Pinene (16.0 mL, 100 mmol, 1.00 equivalent) was added to the solution, followed by methacrylic acid (MAA) (42.0 mL, 500 mmol, 5.00 equiv). Silica-supported trifluoromethanesulfonic acid (7.5% w/w TfOH, 2.00 g, 1.00 mmol, 0.01 equiv) was then added to the reaction mixture, which was stirred at 20 °C for 20 h. The reaction mixture was then filtered under gravity. Organics were washed with DCM (2 × 100 mL). The solution was then evaporated to a quarter of the original volume. The mixture was washed with 1 M aqueous sodium hydroxide solution (500 mL), and then, the aqueous layer was washed with DCM (100 mL). The organic fractions were combined and washed with brine (500 mL) and then dried (Na_2_SO_4_). The resulting mixture was filtered under gravity, and the solvent was removed under reduced pressure to yield the crude mixture. The crude product was then purified using silica gel flash chromatography (eluent mixture of EA in petroleum ether, gradient of 0–5%) to yield a mixture of isobornyl–bornyl methacrylate as a yellow oil (7.83 g, 35%).

Data collected for the mixture of isobornyl–bornyl methacrylate: δH (400 MHz, CDCl_3_, values in ppm): 6.13 (d, *J* = 5.0, 1.9, 1.0 Hz, 1H), 6.10–6.04 (m, 3H), 5.57–5.54 (m, 1H), 5.53 (t, *J* = 1.6 Hz, 3H), 4.93 (ddd, *J* = 9.9, 3.5, 2.2 Hz, 1H), 4.73 (dd, *J* = 7.7, 3.4 Hz, 2H), 2.47–2.34 (m, 1H), 1.99–1.96 (m, 3H), 1.95–1.92 (m, 8H), 1.89–1.79 (m, 5H), 1.79–1.73 (m, 6H), 1.74–1.68 (m, 4H), 1.62–1.54 (m, 4H), 1.02 (s, 8H), 0.90 (s, 4H), 0.88 (s, 8H), 0.86 (s, 11H). δC (100 MHz, CDCl_3_, values in ppm): 166.88, 136.87, 124.91, 86.30, 81.17, 80.12, 48.84, 46.93, 45.06, 38.86, 33.71, 27.05, 20.11, 19.91, 18.32, 11.48. ν_max_ (cm^–1^): 2954 (C–H), 2874 (C–H), 1715 (C=O), 1638 (C=C), 1453, 1306, 1158, 1053.

#### 2.3.2 Synthesis of α-Terpinyl Methacrylate *via* Esterification on a Small Scale

A dried flask was charged with dry tetrahydrofuran (THF) (20 mL) under an inert argon atmosphere. α-Terpineol (0.83 mL, 5.00 mmol) was added with stirring, and the resulting mixture was cooled to 0 °C (water/ice bath). *n*-Butyllithium (2.366 M in hexanes, 2.22 mL, 5.25 mmol) was added to the mixture over 5 min, and the resulting mixture was stirred at 0 °C for 30 min. Methacryloyl chloride (0.52 mL, 5.25 mmol) was then added over a period of 5 min, and the resulting mixture was stirred at 0 °C for 1 h. The reaction mixture was then allowed to warm to room temperature and was stirred for a further 2.5 h. After reaction completion had been indicated by thin layer chromatography (TLC), the reaction mixture was quenched (saturated aqueous sodium bicarbonate, 30 mL). Organics were extracted into diethyl ether (3 × 20 mL). The organic fractions were combined and washed with brine (25 mL) and then dried (MgSO_4_). The resulting mixture was filtered under gravity, and the solvent was removed under reduced pressure to yield the crude product. 151 purifications were carried out using silica gel flash chromatography (eluent of 0–10% EA in petroleum ether) to yield the desired product as a pale-yellow oil (759 mg, 68%).

Data collected for 2-(4-methylcyclohex-3-en-1-yl)propan-2-yl methacrylate: δH (400 MHz, CDCl_3_, values in ppm): 5.94 (1H, dq, *J* = 1.9, 1.0 Hz), 5.47 (1H, app qu, *J* = 1.6 Hz), 5.41–5.35 (1H, m, H-1), 2.09–1.95 (4H, m), 1.94–1.78 (2H, m), 1.90 (3H, dd, *J* = 1.6, 1.0 Hz), 1.65 (3H, m), 1.50 (3H, s), 1.47 (3H, s), 1.35 (1H, app qd, *J* = 12.2, 5.6 Hz); δC (100 MHz, CDCl_3_, values in ppm): 166.6, 138.2, 133.9, 124.2, 120.2, 84.7, 43.1, 30.9, 26.5, 24.1, 23.5, 23.4, 23.3, 18.4. ν_max_ (cm^–1^): 2927 (C–H), 1710 (C=O), 1637 (C=C), 1450, 1329, 1174, 917, 799.

#### 2.3.3 Synthesis of α-Terpinyl Methacrylate *via* Esterification on a Large Scale

α-Terpineol 100 mL (93 g, 0.603 mol) was added to a 500 mL flask which was secured inside a half-full water bath. A thermometer and Ar inlet were connected through a single rubber septum. A mechanical stirrer and a dropping funnel were also connected to the flask. The dropping funnel was sealed with a rubber septum. High vacuum was applied through the Ar inlet for 5 min to degas the α-terpineol. The reactor was backfilled with Ar. A silicone bubble valve was connected through the dropping funnel’s rubber septum, and a slow flow of Ar (1–3 mL/s) was maintained through the reactor. 250 mL of *n*-BuLi (2.5 M in hexane, 0.625 mol) was loaded into the dropping funnel *via* a cannula. Ice was added to the water bath until it was full. *n*-BuLi was added dropwise to the α-terpineol, with vigorous stirring, at a rate slow enough to maintain the temperature of the reaction mixture below 40 °C. Once all the *n*-BuLi was added, the reaction mixture was left to react for an additional 10 min. The dropping funnel was removed and reattached to a 100 mL round bottom flask, while the open neck on the reactor was sealed with a rubber septum. The α-terpineol alkoxide solution was transferred to the dropping funnel *via* cannula techniques. Once transferred, the cannula was removed, and the dropping funnel was reconnected to the 500 mL three-neck round bottom flask. 75 mL of methacryloyl chloride (80.25 g, 0.768 mol) was loaded into the 500 mL flask. 60 mL of THF was added while stirring vigorously. The ice bath was topped with ice, and the α-terpineol alkoxide solution was added dropwise to the methacryloyl chloride solution at a slow enough rate to maintain the reaction temperature below 40 °C. Once all the α-terpineol alkoxide solution was added, the ice bath was removed, and the reaction mixture was left to react for an additional 30 min. The reaction mixture was transferred to a 500 mL separating funnel and washed with 2 × 50 mL of 1 M NaHCO_3_ followed by 2 × 50 mL of deionized (DI) water and 2 × 50 mL of NaCl brine. The organic phase was isolated and dried with MgSO_4_ before removing the volatile solvents *via* a rotary evaporator. The residue was fractionally distilled under vacuum (product B.P. = 88–90 °C at pressures below 1 mbar).

Data collected for 2-(4-methylcyclohex-3-en-1-yl)propan-2-yl methacrylate: see the section mentioned above.

#### 2.3.4 Synthesis of Bornyl Methacrylate

(−)-Borneol (9.87 mL, 64.8 mmol) was dissolved in DCM (500 mL) and cooled to 0 °C. Triethylamine (20 mL, 123 mmol) and methacryloyl chloride (9.5 mL, 97.2 mmol) were then added dropwise, and the reaction mixture was stirred for 24 h during which time it warmed to room temperature. The reaction was monitored by TLC (9:1 hexane/EA). After completion, the reaction was quenched by addition of saturated aqueous solution of NaHCO_3_ (1 × 200 mL). The aqueous phase was extracted with DCM (3 × 200 mL), and the combined organic layers were washed with brine (1 × 200 mL) and dried with MgSO_4_, and the solvent was evaporated in *vacuo.* The resulting residue was re-dissolved in DCM, and the solution was centrifuged. The supernatant was collected and concentrated, producing an orange liquid (71%). ^1^H NMR and ^13^C NMR spectroscopic analysis was performed on the pure sample to establish the actual monomer ratio of the final copolymer composition.

Data collected for bornyl methacrylate: δH (400 MHz, CDCl_3_, values in ppm): 6.13 (1H, s), 5.56 (1H, p, *J* = 1.6 Hz), 4.94 (1H, ddd, *J* = 9.9, 3.5, 2.2 Hz), 2.41 (1H, dddd, *J* = 13.6, 9.9, 4.7, 3.3 Hz), 2.05–1.99 (1H, m), 1.99–1.96 (3H, m), 1.83–1.74 (1H, m), 1.72 (1H, t, *J* = 4.5 Hz), 1.40–1.23 (2H, m), 1.08–1.00 (1H, m), 0.95 (3H, s), 0.91 (3H, s), 0.87 (3H, s). δC (100 MHz, CDCl_3_, values in ppm): 167.74, 136.90, 124.94, 80.16, 53.44, 48.94, 47.78, 44.95, 36.85, 28.05, 27.26, 19.71, 18.89, 18.36, 13.55. ν_max_ (cm^–1^): 2954 (C–H), 2880 (C–H), 1715 (C=O), 1638 (C=C), 1453, 1325, 1295, 1157, 1045.

### 2.4 Polymerization Synthetic Procedures

#### 2.4.1 Polymerization *via* Thiol-Mediated Free Radical Polymerization

The protocol for the synthesis of the homopolymers and copolymers *via* thiol-mediated free radical polymerization was as follows: The appropriate quantities of the monomers were introduced into the required volume of cyclohexanone with stirring, such that a 1:3 v/v ratio mixture was achieved. The thiol chain transfer agent (CTA) benzyl mercaptan (BzSH) was added at a concentration of 3% mol with respect to the monomers. The initiator azobisisobutyronitrile (AIBN) (0.5% wt/wt with respect to the monomers) was dissolved in cyclohexanone and degassed separately prior to being added to the reaction mixture. Finally, the reaction vessel and the AIBN solution were degassed in an ice bucket by purging with argon using a standard Schlenk line for at least 1 h. To commence the reaction, the temperature was raised up to 75 °C in an oil bath and was allowed to continue for 18 h with continual stirring. Polymer purification was conducted *via* precipitation into an excess of methanol. The typical non-solvent:reaction medium ratio was 5:1 v/v in order to enhance the precipitation process, and finally, the precipitated materials were collected in a vial and left in a vacuum oven at 25 °C for at least 24 h.

^1^H NMR spectroscopic analysis was performed on the crude polymerization solution to determine polymer conversion and separately on the precipitate to establish the actual monomer ratio of the final copolymer composition. To evaluate the molecular weight of the materials, the purified samples were dissolved in high-performance liquid chromatography (HPLC)-grade THF for gel permeation chromatography (GPC) analysis. All the spectral data presented are collected at 400 MHz in CDCl_3_, and values are quoted as δH ppm.

### 2.5 Chemical Characterization

#### 2.5.1 Nuclear Magnetic Resonance

Nuclear magnetic resonance (NMR) spectra were recorded at 25 °C using Bruker AV400 and AV3400 spectrometers (400 MHz) using deuterated solvents. Chemical shifts were assigned in parts per million (ppm). ^1^H NMR and ^13^C NMR chemical shifts (δH, δC) are reported with the shift of CDCl_3_ (δH = 7.26 ppm and δC = 77.0 ppm, respectively). Samples were dissolved in deuterated chloroform (CDCl_3_) to which chemical shifts are referenced to (residual chloroform at 7.26 ppm). MestReNova 14.2.1 copyright 2021 (Mestrelab Research S. L.) was used for analyzing the spectra.

#### 2.5.2 Fourier-Transform Infrared Spectroscopy

Spectra were recorded with an attenuated total reflection Cary 630 FTIR spectrophotometer (Agilent Technologies, Santa Clara, CA). 32 interferograms were recorded for each spectrum, with a resolution of 4 cm^–1^, in the range 4000–650 cm^–1^. Infrared (IR) spectra were analyzed using SpectraGryph1.2 software.

#### 2.5.3 Gel Permeation Chromatography

GPC analysis, in this project, has been performed by using an Agilent 1260 Infinity instrument equipped with a double detector with the light scattering configuration. Two mixed C columns at 35 °C were employed, using THF as the mobile phase with a flow rate of 1 mL/min. GPC samples were prepared in HPLC-grade THF and filtered before injection. Analysis was carried out using Astra software. The number- and weight-average molecular weight (*M*_n_ and *M*_w_) and polydispersity (*D̵*) were calculated using narrow standards of poly(methyl methacrylate) (PMMA) for the calibration curve.

#### 2.5.4 Combined Gas Chromatography and Mass Spectrometry Analysis

Gas chromatography and mass spectrometry analysis (GC–MS) analysis was performed with a Thermo Scientific Trace 1300 gas chromatograph. Samples were prepared in MeOH. Column specifications: (TG-5MS) 30 meter length, 0.32 mm diameter, and 0.25 μm film thickness. The GC oven was held at 50 °C for 1 min, and then, the temperature was increased to 350 °C with a gradient of 25 °C/min and then held at the maximum temperature for a further 2 min. The data was visualized using Chromeleon 7 software.

#### 2.5.5 Dip-Coated Glass Slide Preparation

Glass coverslips (*d*: 130.00 mm, *t*: 0.16–0.19 mm) were oxygen plasma-treated (Diener Plasma) for 10 min using a power of 50 W and an initial oxygen pressure of 0.4 mbar. Treated coverslips were immediately dip-coated with a silicone primer solution (MED1-161, Polymer Systems Technology Limited) in 50% v/v acetone and left to dry for 1 h. Subsequently, primed coverslips were twice dip-coated with a 30 mg/mL DCM solution of each synthesized polymer and left to dry at room temperature for 24 h and 7 days in a vacuum oven (<50 mTorr) at 25 °C.

### 2.6 Physical Characterization

#### 2.6.1 Scanning Electron Microscopy

Scanning electron microscopy (SEM) imaging of the resultant microparticles (MPs) was performed using a JEOL JSM-6060LV; the dried microfluidic-produced particles were sprinkled, using a spatula, onto a double-sided adhesive carbon tape. Prior to SEM analysis, the samples were sputter-coated for 4–5 min at 25 mA with a thin gold layer in an argon atmosphere utilizing a Leica EM SCD005 sputter coater (Leica Microsystems GmbH, Wetzlar, Germany) to give approximately a 25 nm-thick coating.

#### 2.6.2 Time of Flight Secondary-Ion Mass Spectrometry

Surface chemical characterization of the final MPs was performed *via* time of flight secondary-ion MS (ToF-SIMS). MPs were placed onto a poly(hydroxyethyl) methacrylate substrate and subjected to MS using a ToF-SIMS IV (IONTOF GmbH, Münster, Germany) instrument. 500 μm × 500 μm scans were taken with a Bi^3+^ primary ion source. Data were calibrated and analyzed using IonToF software.

### 2.7 Processing Procedures

#### 2.7.1 Microfluidic Processing

Droplet microfluidics was utilized as the method for the production of monodisperse MPs within this project (for the schematic, see Figure S1). The experimental setup consisted of a commercially available hydrophilic 3D glass chip with a channel depth of 100 μm (dolomite), which was assembled in a stainless holder. The geometry chosen to generate the MPs was the flow-focusing junction, and the organic and aqueous phases were pumped with two syringe pumps (Harvard Instrument) and connected to the device *via* polytetrafluoroethylene tubes (0.25 mm internal diameter). A high-speed video camera (Fastcam-1024PCI, Photron Limited), which was mounted on an upright microscope (Olympus, BX-51), was applied to observe the droplet formation. The droplets were collected in a glass vessel with water and illuminated by UV light (wavelength of 365 nm, HAYASHI LA-410UV). The resulting MPs, collected in a glass vessel with DI water, were filtered and left to dry for 1 week in an oven. The microchannels were cleaned in between by employing different organic phases by introducing and flushing with DCM, EA, IPA, and distilled water.

#### 2.7.2 3D Printer System

3D printing was performed using a custom-built drop-on-demand piezoelectric printer system consisting of a moveable XY stage and a Nordson EFD jetting valve fitted with a 3 mL ink reservoir. Polymer inks were prepared in cyclohexanone (35% w/v) and jetted through a 150 μm-diameter nozzle controlled by a unipolar waveform (Scheme S2) adapted to suit the specific fluid properties. Droplets were deposited onto a flexible polyethylene terephthalate substrate (Printed Electronics LTD, 0.75 μm thickness), which was heated to 45 °C. A droplet spacing of 750 μm was used for the printed samples, determined by a measured droplet diameter of 950 μm. The 3D films were printed layer by layer, allowing enough time (∼3 min) between layers for the complete evaporation of the solvent and for the solidification of the previously deposited material. Optical microscope images were then taken of the printed films alongside SEM images to confirm the surface topography.

#### 2.7.3 Polymer Microarray Screening Strategy

To predict and rank the capability of the terpene monomers to prevent bacterial biofilm formation, the α parameter equation was used (eq 1). This assesses which pendant group terpenoid structures were most likely to result in high biofilm resistance, guided by previous analyses.^36,37^ The novel terpene monomers that were synthesized and polymerized in this study were targeted to exhibit α values between −5.49 and 1.1. Consequently, to confirm the performance in the bioassay screening of the novel terpene candidates, monomers previously identified for resistance to bacterial attachment were used as controls. These included cyclododecyl methacrylate (CyDMA), ethylene glycol dicyclopentenyl ether acrylate (EGDPEA), *tert*-butyl cyclohexyl methacrylate (*t*BCHA), benzyl methacrylate (BnMA), and hydroxy-3-phenoxypropyl acrylate (HPhOPA). These monomers were chosen for their similarity in the α value and their proven reliability in previous screening.

### 2.8 High-Throughput Biofilm Screening

#### 2.8.1 Polymer Microarray Preparation

Glass slides (25 × 75 mm) were activated by treatment with an oxygen RF plasma with an initial pressure set to 0.3 mbar and power at 100 W (displaying zero reflected power) for 1 min which also served to clean the surface. The coverslips were then silanized using 3-(glycidyloxypropyl) trimethoxysilane in a dried toluene solvent at 50 °C, under argon for 16 h. The silanized glass slides were then rinsed and sonicated in acetone to remove any unbound silane monomer prior to extracting the solvent in a vacuum oven. Epoxy silanized glass slides (25 × 75 mm) were dipped at 9 mm/s into a 200 mL solution of 4% (w/v) poly(hydroxyethyl methacrylate) (pHEMA) dissolved in 95% (v/v) ethanol in water for 2 s. The slides were then retracted at 2 mm/s and allowed to dry under ambient conditions for 5 min. This process was repeated three additional times, after which slides were first dried overnight under ambient conditions and then dried in an oven for 7 days. Microarray printing was carried out using an XYZ3200 pin printing workstation on a BioDot contact printer (CA, USA) and four 946MP6B slotted metal pins (Arrayit, USA) with a tip diameter of 220 μm. This arrangement was used to transfer approximately 2 nL of a mixture containing the 1% (w/v) 2,2-dimethoxy-1,2-diphenylethan-1-one initiator dissolved in a 50% w/v monomer in dimethylformamide solution from a chemically resistant polypropylene 384-well plate onto the pHEMA-coated substrate slides. The metal pins and printer pin head were cleaned with plasma for up to 1 h prior to use, a necessary step to prevent pins from getting stuck or blocked during print runs or from transferring contaminants from previous runs. To obtain regular-sized spots with homogeneous diameter, excess monomer solution adsorbed onto the exterior of the steel pin was initially blotted multiple times onto a normal glass surface prior to printing on the substrate. The printing was done under an argon atmosphere at O_2_ < 2000 ppm, 27 °C, and 30% relative humidity. The freshly printed arrays with 24 materials were vacuum-extracted at <50 mTorr for 1 week to remove the unpolymerized monomer and solvent. In the first screening, each printed microarray slide contained eight replicates per polymer spot separated by 8 mm, while in the second screening, each printed microarray contained three replicates per polymer spot.

#### 2.8.2 Bacterial Growth Conditions

The *P. aeruginosa* strain used in this work is PAO1-Washington sub-line, Nottingham collection, tagged with a fluorescent protein [either mCherry (excitation/emission 587/610 nm) or mClover3 (excitation/emission 506/518 nm)].^40^ For biofilm assays, the bacteria were first grown in lysogeny broth (LB) at 37 °C for 18 h, with shaking at 200 rpm. The culture was centrifuged and resuspended in Roswell Park Memorial Institute (RPMI)-1640 medium to an optical density (OD) at 600 nm of 0.01.

#### 2.8.3 Polymer 2D Biological Assay

Microarray slides were UV-sterilized for 10 min before placing them in a Petri dish containing RPMI-1640 medium and then inoculated with *P. aeruginosa* and incubated for 24 h at 37 °C with shaking at 60 rpm. Control microarray glass slides were also incubated under the same conditions but without bacteria. After incubation, slides were twice rinsed with phosphate buffered saline at room temperature for 5 min and then with distilled water for 5 min. Scale-up experiments were conducted as follows: Glass coverslips coated by the different polymer hits were UV-sterilized and incubated with bacteria at 37 °C with 60 rpm shaking for 24 h. Biomass was quantified using confocal fluorescence microscopy and image analysis. Fluorescence images were taken for both control and treated slides using a GenePix Autoloader 4200AL (Molecular Devices, US) scanner. Once an image had been acquired, the data was extracted using GenePix Pro 6 software and analyzed in Microsoft Excel. The fluorescence value, which correlates with biofilm biomass on the polymer surface, was obtained by subtracting the fluorescence signal acquired from the attached bacteria on the polymer spots and the fluorescence of the corresponding polymer spot on the control slide. To ensure reproducibility and to filter out background noise, a value was accepted if it was larger than three standard deviations of fluorescence output from control slides; else, the value was classified as below the limit of detection (LOD). The polymer array and coated coverslips were examined using a Carl Zeiss LSM 700 laser scanning confocal microscope fitted with 555 nm excitation lasers and a 5×/NA 0.3 objective. Images were acquired using ZEN 2009 imaging software (Carl Zeiss). Biofilm biomass was quantified using ImageJ 1.44 software (National Institutes of Health, USA) and Comstat2.^41^

#### 2.8.4 Bacterial Cell Viability Assay

RPMI medium was conditioned by incubation with the polymer-coated coverslips for 5 days. The conditioned medium samples were then inoculated with *P. aeruginosa* and grown to a stationary phase at 37 °C for 16 h. Cell suspensions were serial-diluted down to 10^–8^ cells/mL, and 10 μL was spotted onto LB agar and incubated overnight, and the colonies were manually counted. Cell numbers were expressed as log_10_ CFU/mL.

#### 2.8.5 Statistical Analysis

Statistical data analysis (as stated in the text) was performed using Microsoft Excel software, considering for the first 2D microarray screening *n* = 16 *N* = 2, for the second screening, *n* = 5 *N* = 1, and for the scale-up experiments, a minimum of three independent replicate values (*n* = 3 *N* = 1). An LOD was applied to the data, such that if fluorescence data was less than 3 times the standard deviation of a measurement, it was given a value of 0. All graphs show error bars representing the standard error of the mean.

## 3 Results and Discussion

As part of a program investigating differentiated routes to produce novel, molecularly differentiated (meth)acrylates from terpene raw materials, a range of bespoke monomers and synthetic pathways had been developed by the authors.^9,12,38,42,43^ This library of (meth)acrylate terpene-based monomers and their associated synthesis routes was investigated in this work for their ability to prevent biofilm development. The Gram-negative pathogen, *P. aeruginosa*, was chosen for the initial biofilm formation assessment using a polymer microarray screening technique since it is frequently found in medical device-associated and nosocomial infections, and its biofilm development has been intensively studied.^30,44^ Due to a range of mechanisms for adaptation, survival, and resistance to multiple classes of antibiotics, infections by *P. aeruginosa* strains can be life-threatening, and it is recognized worldwide as public health threat.^29,31^ The ability to switch from a motile to a sessile biofilm/lifestyle is a survival advantage for many pathogenic bacteria such as *P. aeruginosa*.^45^

**Figure 1:**
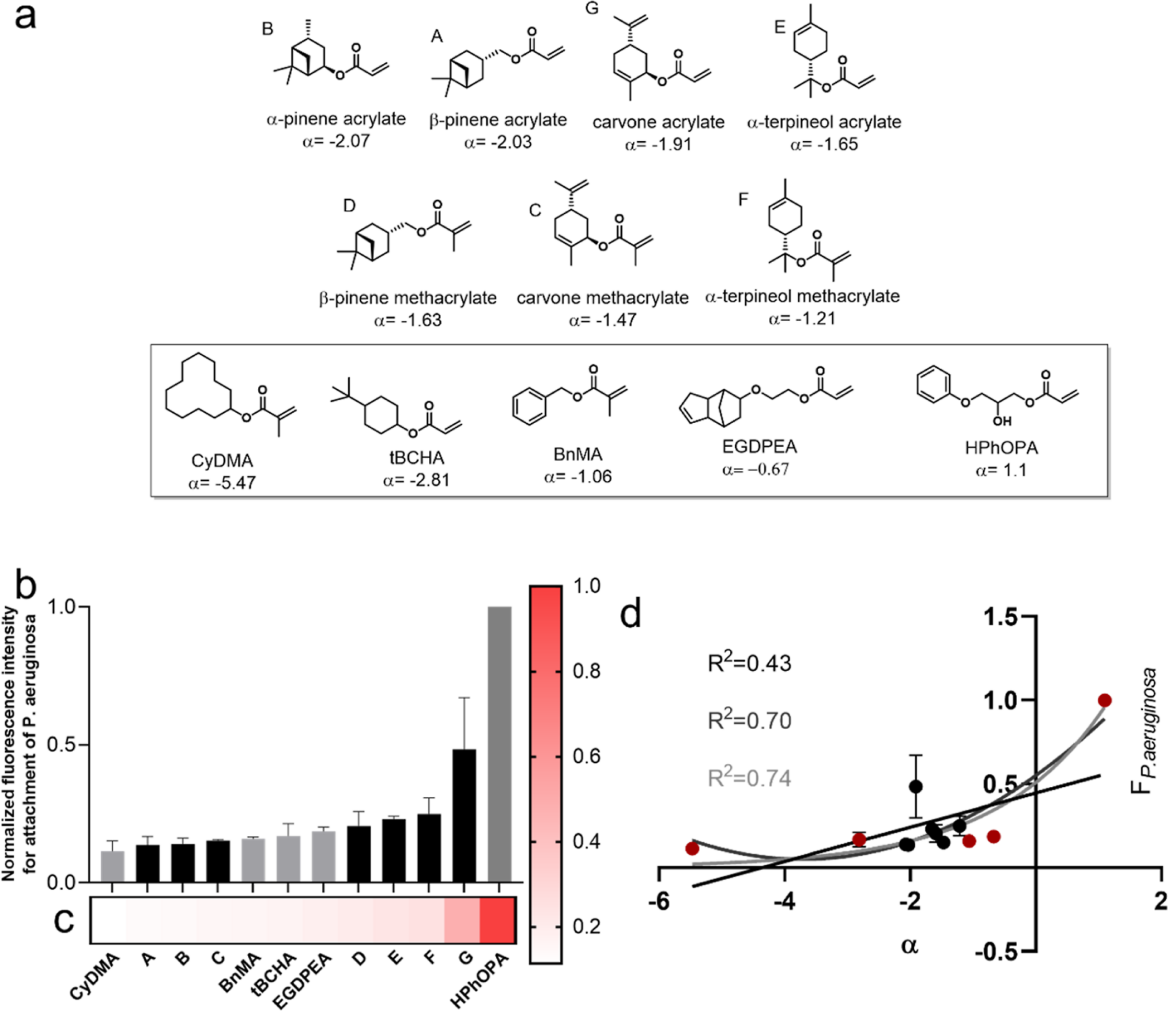
(a) Seven terpene monomers that were initially selected and screened arranged according to their α parameter value from the lowest to the highest (−2.07 to −1.21). Those shown in the bottom row inside the solid box are monomers used as controls in the bioassay. (b) Terpene homopolymer data showing *P. aeruginosa* biofilm formation across a sequential polymer series *n* = 16, *N* = 2. The material screened as α-terpinyl methacrylate (αTMA) is the product obtained from the iron triflate-catalyzed reaction of (−)-α-pinene and MAA. (c) Results from the polymer microarray biological assay with fluorescently tagged *P. aeruginosa*, with monomer identity organized relative to fluorescence. The horizontal scale bar represents the fluorescence value for *P. aeruginosa* biofilm formation (red indicating a high biofilm and white, low). (d) Normalized microarray fluorescence intensity measured for *P. aeruginosa* plotted against the monomer α value. The black symbols and the red symbols represent the materials used to build the terpene library and the positive and negative comparators, respectively. The black line is the linear relationship of the data set (*R*^2^ = 0.43). The dark-gray line is the polynomial relationship of the data set (*R*^2^ = 0.70). The light-gray line is the exponential relationship of the data set with α parameter values of interest.

To define whether any of these new candidates represented a precursor to a potential sustainable biofilm-resistant polymer, their molecular structures were subject to analysis using the molecular descriptor “α parameter” developed by the authors.^37^ Prior work with commercially available monomers found that homopolymers that exhibited bacterial attachment resistance were monoacrylate monomers with hydrocarbon pendant groups and α values in the range −5.47––1.06. These included BnMA, *t*BCHA, CyDMA, and EGDPEA which were used as positive comparators in the bioassays (see Figure 1a). Additionally, HPhOPA was selected because of the higher α value (1.1) which correlated with higher bacterial biofilm formation on surfaces coated with this homopolymer and thus acted as a negative, biofilm-supporting comparator.^37^ Out of the full range of the available terpene structures, those identified in Figure 1a exhibited α parameters within the desired range predicted to deliver the bacterial biofilm resistance characteristics (*i.e.,* −5.47 to 1.1).

The biological screening was performed on a glass pHEMA-coated microarray substrate, where materials could be deposited onto a solid in discrete locations in the form of spots, with each deposition providing a unique and parallel datapoint.^46−51^ Prior studies had shown that neither the pHEMA coating nor unreacted monomer leached from the spot had any negative influence on the bioassay results.^52,53^ The monomeric spots were polymerized *via* photo-polymerization, and subsequently, the *P. aeruginosa* attachment was evaluated to allow a rapid evaluation of the biofilm formation of all the polymer spots. A polymer microarray of 15 spots in 3 replicates was produced with 2 groups of monomers. The first group contained monomers from the terpene library, while the second group was formed from the previously screened commercially available monomer controls.

The comparison between the two group of materials validated that all of these terpene-based candidates exhibited some biofilm resistance against *P. aeruginosa*, clearly identifying them as potential “hit” materials. The majority of the novel sustainable polymers displayed average bacterial biofilm resistance that was not statistically different from one of the positive controls and significantly outperformed the negative control polyHPhOPA (Figure 1b,c).

Furthermore, a similar trend to that observed by Dundas, Sanni *et al.* was defined when correlating the bacterial load measured as fluorescence intensity of *P. aeruginosaversus* α.^37^ As illustrated in the previous work, an exponential fit was demonstrated to better describe the relationship between α and the bacterial attachment for the full polymer library data set with the α value between −5.47 and 1.1. A non-linear regression (*i.e*., exponential) *R*^2^ value of 0.74 (Figure 1d) was observed, significantly better than that of the comparative linear (*R*^2^ = 0.43) and the polynomial correlation (*R*^2^ = 0.70).

**Figure 2:**
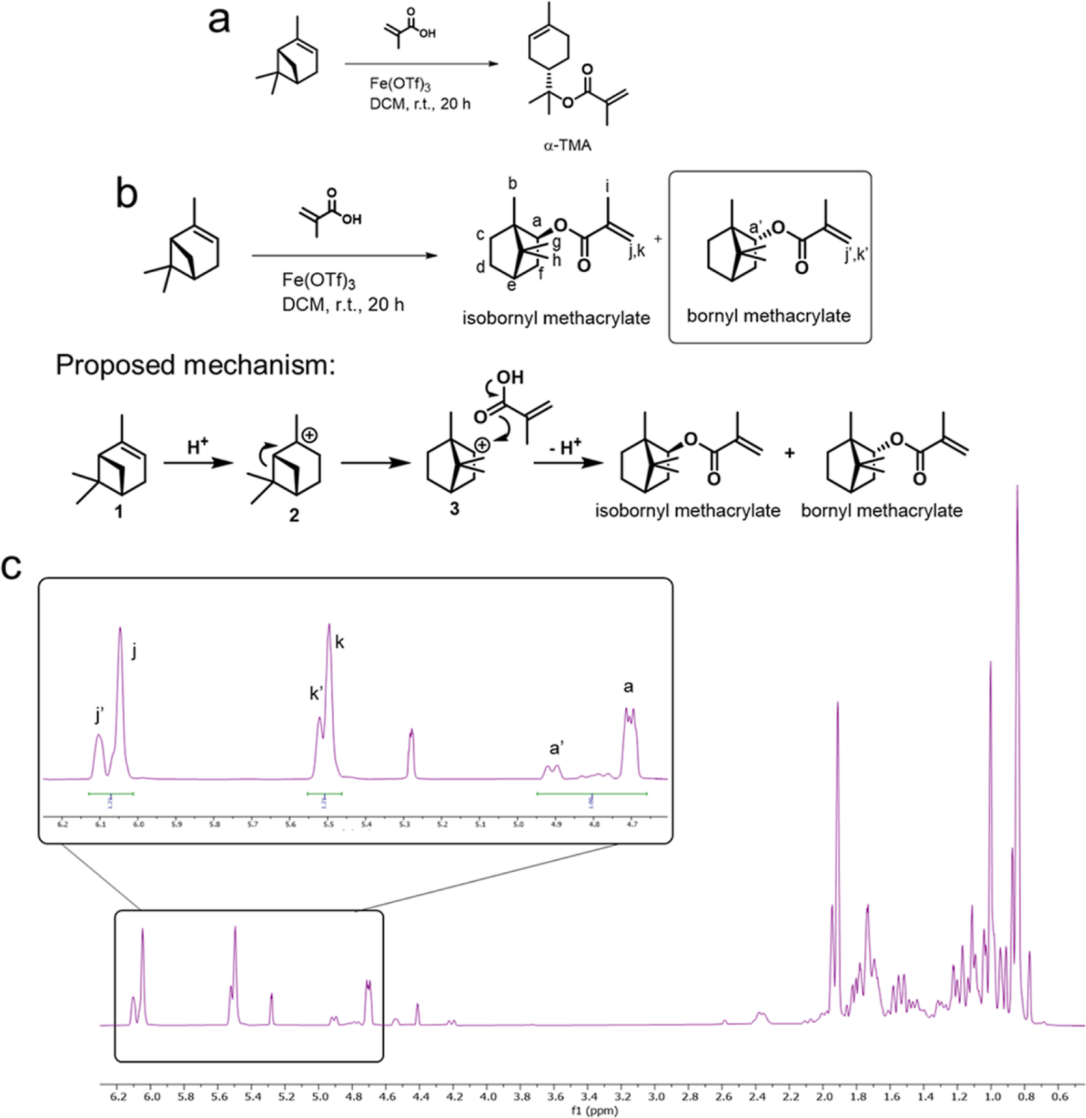
(a) Initial one-step synthesis of αTMA, (b) actual outcome of the one-step αTMA synthesis strategy and the proposed mechanism for the rearrangement that results in the isolation of mixed products, and (c) ^1^H NMR spectrum indicating the mixed products. Integral values are provided for peaks a + a′, j + j′, and k + k′.

A second criterion that was required for the potential larger-scale/commercial exploitation of such sustainable monomers was the synthetic strategy. To generate the candidate monomer, the synthetic route should not be overly complex. The monomers of interest mentioned above, α-pinene (meth)acrylate, β-pinene (meth)acrylate, and carvone (meth)acrylate, were already successfully synthesized with high yield *via* multi-step processes.^38^ These two steps typically involved either hydroboration/oxidation or carbonyl reduction followed by esterification of the resulting alcohol with either (meth)acryloyl chloride or acrylic acid catalyzed by propylphosphonic anhydride (T3P). However, the synthetic pathway for αTMA did have a proposed single-step synthesis starting from the abundant α-pinene and MAA (Figure 2a). The initial element of the sustainability improvement with these monomers was that the pendant group on the monomer was from a sustainable, non-food-competing, and agricultural source, rather than being derived from petrochemicals. However, sustainability is not delivered simply by the sourcing of reagents; the manufacturing route has as much, if not more, influence on the true sustainability of a material. Thus, the focus in the study was also to keep the number of processing stages needed to produce the monomer to a minimum. The pathway for αTMA did have a proposed single-step synthesis starting from the abundant α-pinene and MAA (Figure 2a), and so, the study initially focused on this target system. This strategy was taken into consideration thanks to the relevant recent progress in developing “green” routes to the production of MAA; the state of the art in this field has very recently been summarized in a review by Lebeau *et al*.^54^

There has been significant interest in alkene hydrofunctionalization reactions in the recent literature, as detailed in several reviews.^55,56^ Of particular interest to this study was the use of Fe(III) triflate to achieve inter-molecular hydrofunctionalization reactions.^57,58^ Specifically, it was hypothesized that Fe(III) triflate may catalyze the coupling of readily available terpenes with MAA and so provide a single-step method to access terpene-derived methacrylates. Thus, (−)-α-pinene and MAA were employed in an attempted synthesis of αTMA, as shown in Figure 1a.

On the small laboratory scale, the use of Fe(III)triflate was demonstrated to deliver an approximately 22% yield of a mixture of methacrylate-containing compounds after chromatographic separation. Interestingly, the ^1^H NMR spectrum (Figure 2c) contained a series of peaks not related to the resonances expected for the targeted αTMA product. In particular, the 4 to 5 and 5.5 to 6.3 ppm regions contained signals corresponding to two distinct methacrylate derivatives. Subsequent 2D-heteronuclear single-quantum coherence (HSQC) NMR analysis (see Figure S2) was used to qualitatively evaluate the nature of these peaks. The HSQC spectrum of the purified product confirmed that the peak set at 4–5 ppm represented tertiary carbons corresponding to (CH) groups next to heteroatoms (at around 70–80 ppm). Meanwhile, the 5.5 ppm and 6.3 ppm resonances were identified as corresponding to alkenyl protons from methacrylate functionalities.

Further investigation of the ^1^H (Figure 1), ^13^C NMR (Figure S3), and HSQC (Figure S2) spectra revealed that the crude methacrylate monomer product obtained *via* this proposed one-step strategy contained a mixture of bornyl and isobornyl methacrylate as the major components (see Figure 2b, now referred to as BoMA and *i*BoMA, respectively). The mechanism proposed for the formation of BoMA–*i*BoMA is shown in Figure 2b. Based on prior literature reports, it was proposed that under the reaction conditions applied, α-pinene was protonated by the acid catalyst (**1**) to give the pinyl cation (**2**). A well-established Wagner–Meerwein rearrangement would then result in the formation of the comparatively stable bornyl cation (**3**).^59−61^ Finally, trapping (**3**) with MAA from either the exo- or endo-face would lead to the isobornyl methacrylate or bornyl methacrylate, respectively.

In order to confirm this assignment, spectroscopic analysis of pure BoMA and *i*BoMA samples was performed (Figures S4 and S5). While *i*BoMA is commercially available, BoMA was synthesized following a methacryloyl chloride route methodology (see the Materials and Methods section). Both ^1^H NMR and ^13^C NMR analyses indicated that *i*BoMA was the majority component, present in a ratio of 60:40 mol/mol with its bornyl isomer. This assignment was confirmed by GC–MS analysis *via* comparison of the data collected from authentic samples of *i*BoMA and BoMA, Figures S6–S8.

**Figure 3:**
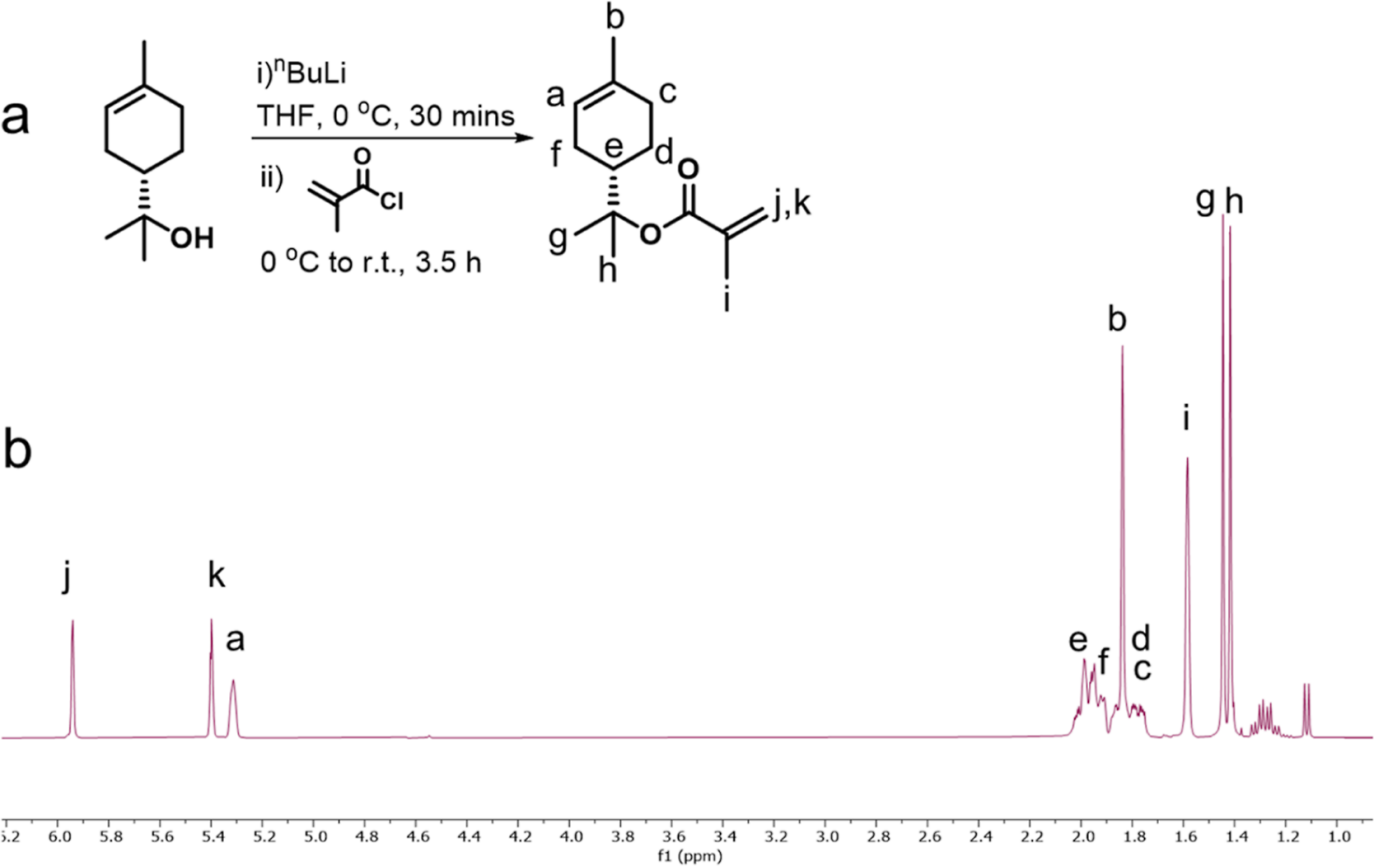
(a) Proposed αTMA synthesis route from α-terpineol by esterification using methacryloyl chloride. (b) ^1^H NMR of αTMA isolated from this preparative route with assigned peaks.

Furthermore, this NMR and GC–MS analysis demonstrated that the target product, αTMA, was not detected in the reaction mixture (Figure S10). Thus, the development of a new, more efficient one-step esterification strategy to synthesize αTMA was investigated. The proposed synthetic route would produce the targeted αTMA structure from α-terpineol by deprotonation and subsequent esterification using methacryloyl chloride. Initial small-scale exploratory (1 g) reactions were shown to generate the desired product in 68% yield. ^1^H NMR of αTMA confirmed that the product exhibited the expected structure (Figure 3).

The characteristic side chain (C=C) signal is clearly identified with a singlet at 5.35 ppm. In addition, the (CH_3_) group peaks, along the sidechain, were observed in a high field at a chemical shift of 1.8, 1.5, and 1.4 ppm. The HSQC spectrum also confirmed the structure of the monomer (Figure S9). GC–MS analysis of a sample from the reaction product of α-terpineol and methacryloyl chloride (Figure S10) also confirmed that the product material did not contain either *i*BoMA or BoMA.

This easy one-pot reaction protocol clearly outperformed the Fe(III) triflate synthesis route by producing the targeted monomer in high yield and avoiding unwanted rearrangements which lead to new terpenoid species. Furthermore, the one-pot synthesis developed in this study was shown to be easily and successfully translatable to the 100 g scale. This paved the way to large-scale production of terpene-hybrid polymerizable monomers for the bio-instructive assays and microfluidic processing trials.

In light of this deeper study on the chemical nature and composition of the products obtained *via* the two different proposed synthetic strategies, a further bacterial attachment assay was conducted to compare biofilm formation on (a) the mixed products (*i*BoMA–BoMA) obtained *via* hydrofunctionalization of α-pinene with MAA and catalyzed by Fe(III) triflate; (b) authentic *i*BoMA and BoMA; (c) copolymers between *i*BoMA and BoMA (v/v ratio 0:100, 10:90, 30:70, 50:50, 70:30, 90:10, and 100:0), and (d) αTMA synthesized *via* esterification with methacryloyl chloride. The biofilm-resistant control in this study was *i*BoMA, and the pro-attachment was HPhOPA.^36,48,62^

**Figure 4:**
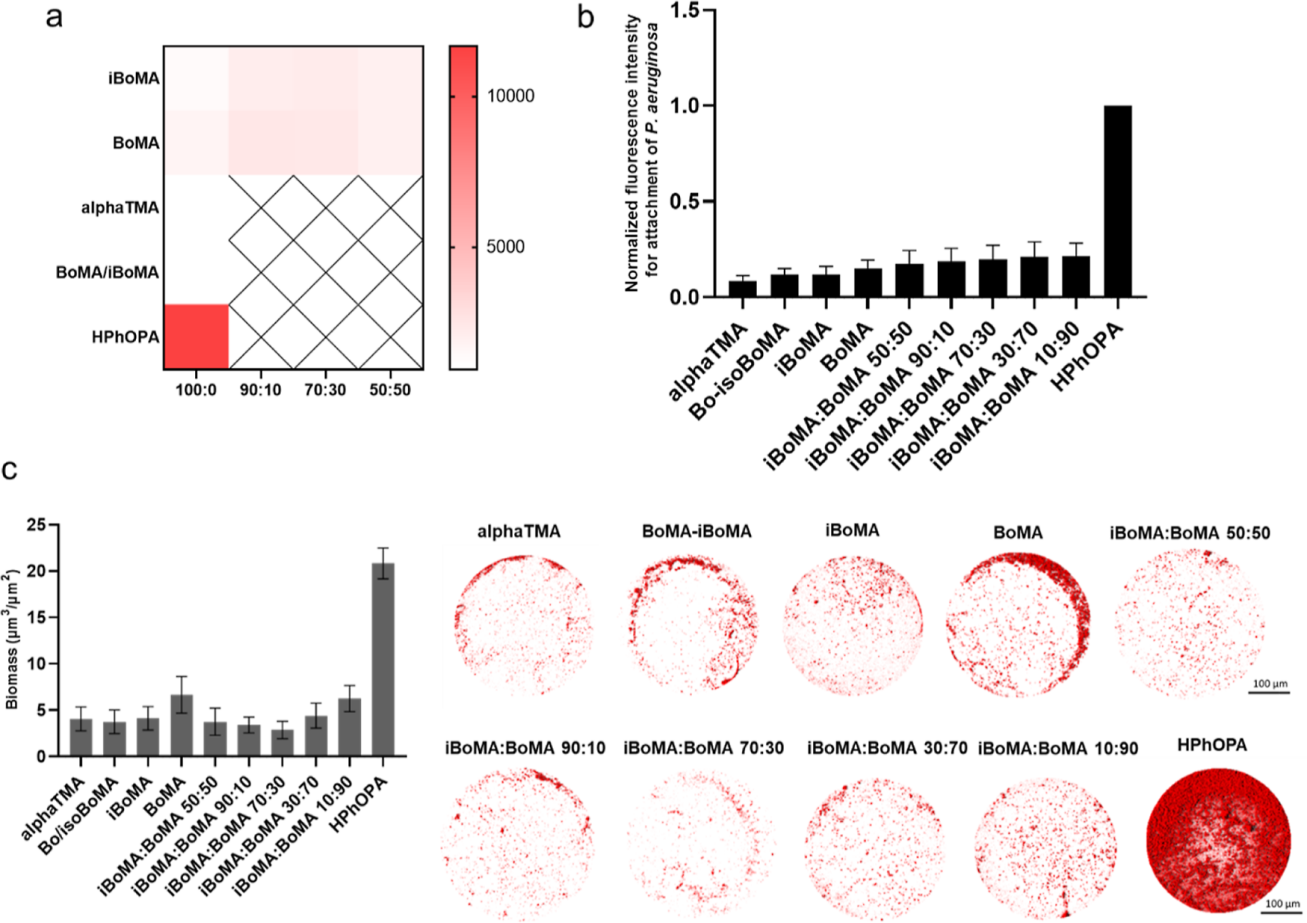
(a) Results from the polymer microarray biofilm assay with mCherry-tagged *P. aeruginosa*. Monomer identity is organized into increasing fluorescence response for both the synthesized products and mixtures of commercially purchased *i*BoMA and BoMA. The center square is the fluorescence value for the *P. aeruginosa* biofilm (red indicating a high bacterial biofilm and white, a low biofilm). The hatched area indicates mixing combination that was not investigated. (b) Terpene homopolymer and copolymer data showing the *P. aeruginosa* biofilm across a sequential polymer series *n* = 5, *N* = 1. The material screened as αTMA is the product obtained from the esterification of α-terpineol with BuLi, while BoMA–*i*BoMA is the product of the Fe(III) triflate-catalyzed reaction of (−)-α-pinene and MAA. (c) Confocal microscopy images of mCherry-tagged *P. aeruginosa* for the 3D representation and quantification of the biomass biofilms on the polymer spots.

Figure 4b indicates that all the homo- and copolymers tested exhibited a similar low level of bacterial biofilm colonization to the positive comparator (*i*BoMA). Furthermore, the pure α-terpinyl material synthesized *via* the methacryloyl chloride route nominally demonstrated the best resistance. However, this response was very similar to that of the material produced from the Fe(III) triflate chemistry. The purposely made mixtures of *i*BoMA and BoMA all performed at a similar level suggesting that the bornyl and isobornyl materials exhibit similar levels of resistance to *P. aeruginosa* biofilm formation. This was supported by the observation that the pure BoMA and *i*BoMA performance was not statistically different.

A similar trend was observed when the quantification of biofilm biomass was performed on the spots of these materials (Figure 4c). The pure α-terpinyl material synthesized *via* the methacryloyl chloride route and the material produced from the Fe(III) triflate chemistry showed the lowest biomass contamination compared to the commercially available *i*BoMa and BoMA and their mixtures. This confirmed that the successful one-pot reaction protocols, reported in this work, can produce materials from sustainable and renewable sources with anti-biofilm properties able to successfully replace the correspondent petrol-chemical-derived materials.

Therefore, as the new candidates had to be highly resistant to *P. aeruginosa* biofilm formation, they were used as reagents in thiol-mediated free radical co-polymerizations to generate polymeric surfactants (surfmers) for use in microfluidic processing. A thiol CTA was employed to control the polymerization to produce relatively low-molecular weight homopolymers, random copolymers, and comb graft structures *via* copolymerization with methoxy-poly(ethylene glycol) (PEG) methacrylates. This technique was selected due to the robustness of thiols as CTAs for both (a) a broad range of monomers containing differing functionalities in their pendant groups and (b) their utility in larger-/commercial-scale reactions. In this study, BzSH was used as the model CTA. One of the key aspects of this study is to derive bio-instructive particles. In order to enable the microfluidic process to successfully produce these monodisperse particles *via* the routes that will ensure that the bio-instructive species is on the surface of the particles, the surfmer had to be dissolved in the dispersed phase. The dispersed phase also needed to have a relatively low viscosity in order to be separated into micelles at the impingement point within the microfluidic chip. To achieve this low viscosity, the molecular weights and polydispersity indexes of the polymers produced were kept low. The complete range of polymerizations conducted is shown in Table 1.

**Table 1:** Polymerization and Material Property Data for the Homo- and Co-Polymers of Isobornyl, Bornyl, α-Terpinyl, and Methoxy-PEG Methacrylates and a Sample of the Product from the Fe(III) Triflate Synthesis

entry	material	*M*_n_^a^(g/mol)	*M*_w_^a^(g/mol)	*D̵*^a^	conv^b^ (%)
1	poly(*i*BoMA)	5300	7600	1.44	>90
2	poly(BoMA)	6200	9700	1.57	>90
3	poly(αTMA)	7100	16,500	2.33	>90
4	*i*BoMA-*co*-BoMA	7300	11,400	1.57	>90
5	poly(BoMA–*i*BoMA)_mix_	40,000	68,000	1.67	>90
6	(BoMA–*i*BoMA)_mix_-*co*-*m*PEGMA_300_	24,900	47,100	1.89	>90

a Measured by GPC against narrow PMMA standards.

b Calculated by ^1^H NMR.

Homopolymerization of the following monomers was performed: *i*BoMA (entry 1), BoMA (entry 2), and αTMA (entry 3). In addition, two copolymers were added to this palette of homopolymers: *i*BoMA-*co*-BoMA (polymerized from pure commercially sourced monomers mixed in a molar ratio of 60:40 mol/mol) and poly(BoMA–*i*BoMA)_mix_ [polymerized from the column-purified product from the Fe(III) triflate synthesis]. Meanwhile, to determine the suitability of the new bio-derived monomers to have utility as the basis of novel polymeric surfactants, the mixture of BoMA–*i*BoMA, obtained from the Fe(III) triflate synthesis, was copolymerized with poly(ethylene glycol) methyl ether methacrylate (*m*PEGMA_300_) according to a similar procedure showed by the authors.^63^

The data in Table 1 demonstrate that use of BzSH successfully resulted in the synthesis of both homopolymers and copolymers with molecular weights generally lower than 10,000 g/mol. A small concentration of this CTA (1% mol with respect to the monomer) enabled the control of each polymer resulting in relatively low dispersity (*i.e.*, *D̵* <2.00). However, the polymerization of poly(BoMA–*i*BoMA)_mix_ did not follow this trend. Contrary to the rest of the copolymerizations, it resulted in a quite-high-molecular weight product (40,000 g/mol) indicating that the impurities of this material might negatively affect the chain transfer mechanism of the CTA and/or significantly reduce the number of radicals that are available to conduct the polymerization. This conclusion was supported by the observation that as the concentration in the polymerization mixture of the BoMA–*i*BoMA monomer was reduced in the surfactant synthesis by the inclusion of the *m*PEGMA comonomer, the level of control over the polymerization increased.

**Figure 5:**
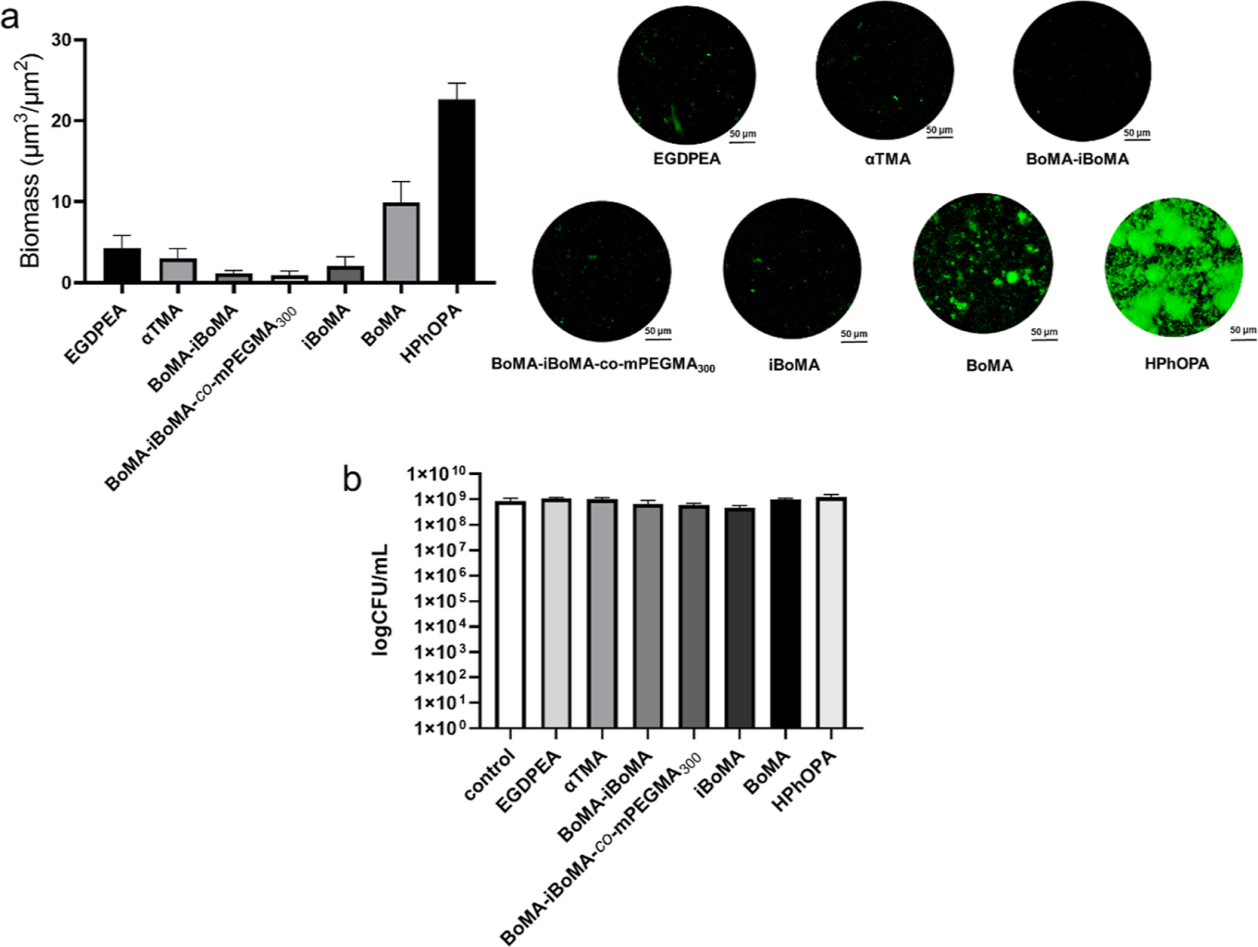
(a) Results from scale-up biofilm assay with *P. aeruginosa* (tagged with the fluorescent protein mClover3). On the left, biomass biofilm quantification after incubation of *P. aeruginosa* on glass coverslips dip-coated by the polymer candidates is shown (*n* = 4, *N* = 1); on the right, representative confocal microscopy images of biofilms grown on the surface of the polymer-coated coverslips are shown. Scale bar, 50 μm. (b) Bacterial cell viability assay obtained after inoculation of *P. aeruginosa* in conditioned medium (by incubation with polymer-coated coverslips for 5 days). Cell numbers are expressed as log_10_ CFU/mL obtained by manual colony counting after cell suspensions were spotted onto LB agar plates (*n* = 3, *N* = 1). The control used was fresh, unconditioned RPMI medium. Cell population density (OD_600_) was recorded every 30 min over a period of 17 h or until a stationary phase was reached. The control was fresh unconditioned RPMI medium.

These newly synthesized materials were tested *via* scale-up against *P. aeruginosa* to confirm the results obtained on the microarray biofilm assay. Glass coverslips were dip-coated using solutions containing terpene-based polymers, polyαTMA, poly(BoMA–*i*BoMA)_mix_, BoMA–*i*BoMA-*co*-*m*PEGMA_300_, poly*i*BoMA, and polyBoMA, to form viable coatings and then used to study their ability to prevent *P. aeruginosa* biofilm formation (Figure 5a). In the same assay, the performance of these novel sustainable materials was compared with that of acrylate homopolymer controls, polyEGDPEA (positive) and polyHPhOPA (negative), i.e., materials previously reported in the literature to prevent or enhance bacterial biofilm formation, respectively. Furthermore, in addition to the biofilm assay, we investigated whether any residual soluble component cytotoxic bacteria leach from the polymers. Figure 5b shows that *P. aeruginosa* viability was not affected by growth in medium conditioned for 5 days by incubation with the polymers.

Figure 5a shows a significant reduction of bacterial biomass between the biofilm-supporting control polymer polyHPhOPA and each of the other polymers tested. The biomass values obtained from the polymer samples, synthesized *via* normal laboratory methods, were shown to be consistent with those obtained for the polymer microarray. In particular, polyBoMA showed the lowest biomass values of the terpene hits used in this study. This highlighted that the high-throughput screening is key in the initial proof-of-concept evaluation of new bio-instructive polymer candidates. Finally, further toxicity tests were performed to examine whether any leached materials affected the growth of bacteria. Pre-incubation with the test polymers demonstrated no inhibition or killing of planktonic bacterial growth (Figure 5b).

**Figure 6:**
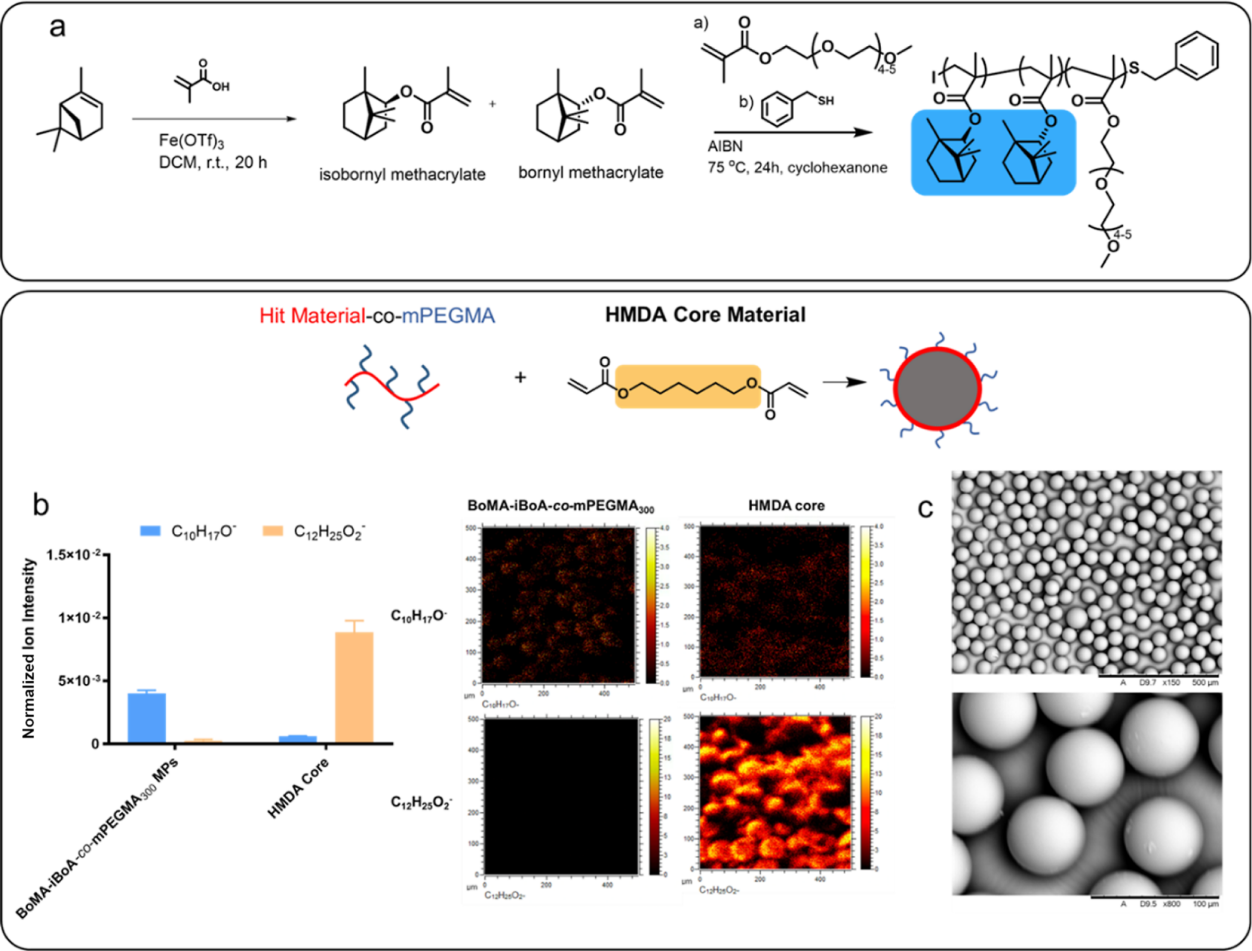
(a) Synthetic pathway describing the different steps required for the synthesis of the monomers and surfmer. (b) ToF-SIMS data showing intensities of two key ions associated with the BoMA–*i*BoMA side chain and HMDA core materials within the surfactant structures (C_10_H_17_^–^—BoMA–*i*BoMA, C1_2_H_25_O_2_^–^—HMDA) where the ions from the structures are circled in blue and orange, respectively. Corresponding ion intensity images for C_10_H_17_O^–^ and C_12_H_25_O_2_^–^ shown in counts per pixel (*n* = 3). (c) SEM images of monodisperse MPs made with the (BoMA–iBoA)_mix_-*co*-*m*PEGMA_300_ surfactant with a core of HMDA and a size of 61 ± 2.68 μm [coefficient of variation (CV) = 4.34%].

To demonstrate that these selected sustainable bioderived monomers could be used as alternatives to bacterial biofilm-resistant petrochemically based monomers, this study also investigated the design optimization of target polymer surfactants (*i.e.*, surfmers) with the terpene monomer. This involved maximizing both the (a) delivery of specific bio-instructive molecules and (b) application to microfluidic processing. As such, this work extended the work reported in prior papers by the authors, where the original petrochemically based monomers were used to synthesis bio-instructive surfmers and then surface active particles which were shown to be biofilm-resistant.^63−66^ Thus, in this study, the preparation of MPs was conducted using the same microfluidic system settings that were utilized in the prior reports; i.e., an O/W droplet flow-focusing chip was used along with a surfactant constructed from randomly polymerized BoMA–*i*BoMA and *m*PEGMA_300_ (Figure 6a). This has given the authors the confidence that these surfmers will also exhibit biofilm resistance in the same way that the petrochemical versions have been reported to exhibit, and a longer-term study of this is now currently underway to explore this aspect.

SEM images (Figure 6c) showed that the MPs generated using O/W droplet microfluidics are perfectly spherical with a diameter of 61.0 ± 2.7 μm which was lower than the orifice width (100 μm), highlighting that the presence of the surfactant controls the surface tension between the two phases. The resulting coefficient of variation (CV) used to measure a quality of the particle size distribution was lower than a 5% CV which is considered a narrow distribution, confirming that these particles are monodisperse. ToF-SIMS analysis was conducted to study the surface chemistry of the MPs produced (Figure 6b).

Data was collected in the negative ion mode in order to detect the unique ion related to the structure (BoMA–BoMA)_mix_-*co*-*m*PEGMA_300_ (C_10_H_17_O^–^) and was compared with that of the HMDA core particles prepared without the aid of the surfactants to demonstrate the difference between the unfunctionalized and functionalized particles. This comparison clearly demonstrated that the surfactant unique ion (C_10_H_17_O^–^) was located at the surface of the MPs; therefore, the presence was demonstrated to ensure that the surface was functionalized with the biologically active material of choice.

**Figure 7:**
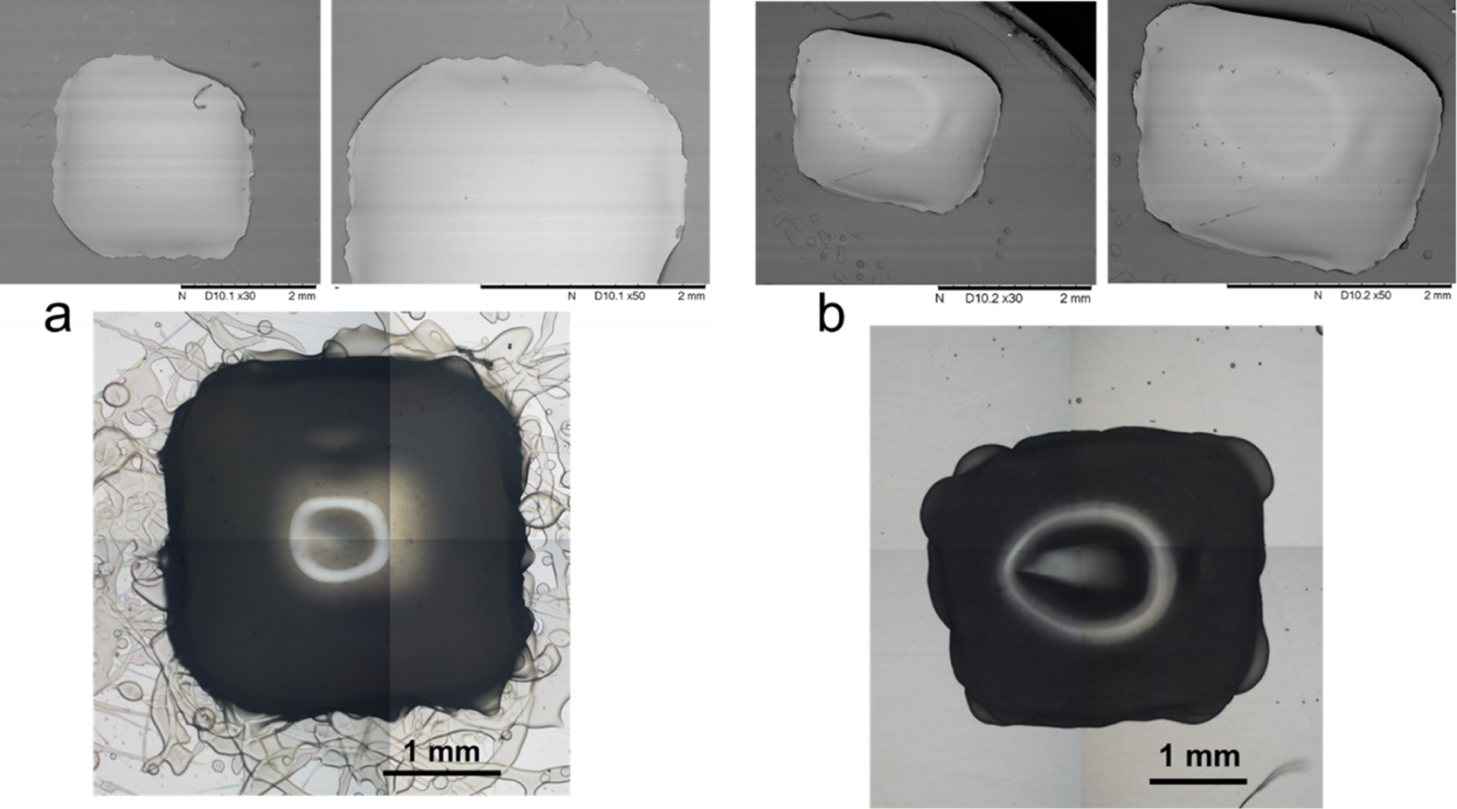
(a) Optical microscope and SEM images (30× and 50×, respectively) of polyαTMA. (b) Optical microscope and SEM images (30× and 50×, respectively) of poly(BoMA–*i*BoMA)_mix_.

In addition to demonstrating the viability of using these new terpene monomers to form bio-active surfactants that were successfully utilized in the manufacture of bio-instructive MPs, we have also explored the possibility of printing these homopolymers using an additive manufacturing (AM) technology to demonstrate that these polymers can be exploited as materials of construction for medical devices. Recent advancements in AM methods have allowed the fabrication of specimen articles with precise and complex structures, opening new frontiers in the application of such materials in medical treatment. In this study, a method known as “valve-based” jetting was demonstrated to successfully enable the processing of the terpene polymers in the form of solvent solution feedstocks.^67−71^ Use of this AM allowed discrete volumes (nL) of the viscous feedstocks to be dispensed on demand to form pre-determined 3D print shapes. Figure 7 contains examples of the successfully printed polyαTMA and poly(BoMA-*i*BoMA)_mix_ cuboid.

Figure 7 shows that it is possible to apply these polymeric inks in AM to form medical devices. However, from both the optical microscopy and SEM images, it can be observed that the surface is not completely smooth, and some edge definition has been lost; thus, further material formulation and process fidelity optimization is still required.

## 4 Conclusions

In conclusion, this work has demonstrated, for the first time, that both (meth)acrylate terpene-based monomers derived from commercially available terpenes can be synthesized by adopting a one-step, one-pot synthetic strategy and that their resulting polymers practically deliver high levels of bacterial anti-attachment performance. Furthermore, rearrangements in the monomer structure during monomer synthesis, due to the multi-functional nature of the terpene alcohol precursors, were shown to be possible, resulting in the isolation of a mixture of terpene monomers. Furthermore, all of these monomer structures, including those present in the mixed product, were predicted to produce polymers that exhibit resistance to bacterial attachment by application of the α parameter. These predictions have been successfully validated *via* high-throughput microbiological screening, adding further validation to the use of this molecular descriptor tool. From this screening, α-TMA was selected as the most promising monomer to upscale due to a combination of its high levels of bio-response and the possibility that it could be synthesized utilizing several potential one-pot synthetic strategies. The targeted α-TMA terpene based monomer was successfully synthesized at larger scale (100 g). The scale-up of this monomer was executed via one of these potential one-pot routes (esterification), while a second produced an *i*BoMA–BoMA mixture (methacryloxylation). Due to the greater simplicity of the monomer synthesis of the latter, it was then used to successfully produce polymeric, comb-graft surfactants *via* copolymerization with a PEGMA comonomer which were subsequently utilized to manufacture bio-instructive particles by multifluidic processing. Thus, this work has demonstrated how sustainably sourced monomers can be straight forwardly synthesized and polymerized to produce highly versatile, bio-responsive materials, both as part of 2D and 3D platforms which are of high utility for different final applications, minimizing the reliance on fossil-based building blocks.

## Supporting Information Available

Mechanism for the synthesis of bornyl methacrylate; schematic of the experimental microfluidic setup; typical unipolar waveform for valve-based jetting; 2D-HSQC NMR spectrum used to qualitatively evaluate the nature of the product mixture obtained from the iron triflate-catalyzed reaction of (−)-α-pinene and MAA; ^13^C NMR spectrum used to qualitatively evaluate the nature of the product mixture obtained from the iron triflate-catalyzed reaction of (−)-α-pinene and MAA; comparison of the ^1^H NMR spectra of the iron triflate synthesized mixture of isobornyl and bornyl methacrylate, pure isobornyl, and pure bornyl methacrylate monomers; comparison of the ^13^C NMR spectra of the iron triflate synthesized mixture of isobornyl and bornyl methacrylate, pure isobornyl, and pure bornyl methacrylate monomers; GC–MS analysis data collected from the reaction product of pinene and MAA; GC–MS analysis data collected from authentic samples of isobornyl methacrylate; GC–MS analysis data collected from authentic samples of bornyl methacrylate; HSQC spectrum which confirmed the structure of the α-TMA monomer; GC–MS analysis data collected from authentic samples of α-TMA; and SEM images of monodisperse MPs made with the (BoMA–iBoA)_mix_-*co*-*m*PEGMA_300_ surfactant with a core of HMDA and size of 61 ± 2.68 μm (CV = 4.34%) (PDF)
