# Supplementary material for: Predictive Molecular Design and Structure–Property Validation of Novel Terpene-Based, Sustainably Sourced Bacterial Biofilm-Resistant Materials
Source: Biomacromolecules. 2023 Jan 4;24(2):576–91. doi: 10.1021/acs.biomac.2c00721 (PMC9930090; doi:10.1021/acs.biomac.2c00721)
Supplement: Supplementary file 1 — bm2c00721_si_001.pdf [file bm2c00721_si_001.pdf]

Predictive Molecular Design and Structure-Property Validation of Novel Terpene Based, Sustainably Sourced Bacterial Biofilm Resistant Materials

Valentina Cuzzucoli Crucitti,[a]* Aleksandar Ilchev,[a] Jonathan C. Moore,[b] Harriet R. Fowler,[b] Jean-Frédéric Dubern,[d] Olutoba Sanni,[c] Xuan Xue,[c] Bethany K. Husband,[a] Adam A. Dundas,[a] Sean Smith,[b] Joni L. Wildman,[a] Vincenzo Taresco,[b] Paul Williams,[d] Morgan R. Alexander,[c] Steven M. Howdle ,[b] Ricky D. Wildman,[a] Robert A. Stockman[b] and Derek J. Irvine[a]*

[a] Centre of Additive Manufacturing, Department of Chemical and Environmental Engineering, University Park, University of Nottingham, Nottingham, NG7 2RD
[b] School of Chemistry, University Park, University of Nottingham, Nottingham, NG7 2RD
[c] Advanced Materials and Healthcare Technologies, School of Pharmacy, University Park, University of Nottingham, Nottingham, NG7 2RD
[d] National Biofilms Innovation Centre, Biodiscovery Institute and School of Life Sciences, University Park, University of Nottingham, Nottingham, NG7 2RD

*Corresponding authors: Valentina.CuzzucoliCrucitti1@nottingham.ac.uk and Derek.Irvine@nottingham.ac.uk

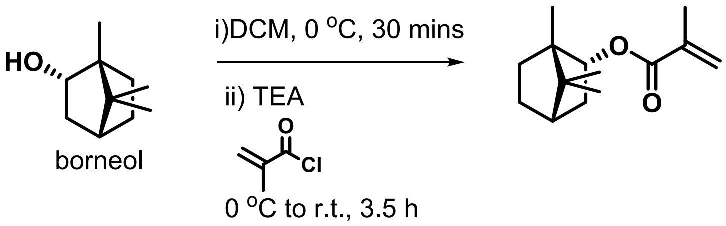

Scheme S1 Mechanism for the synthesis of Bornyl Methacrylate.

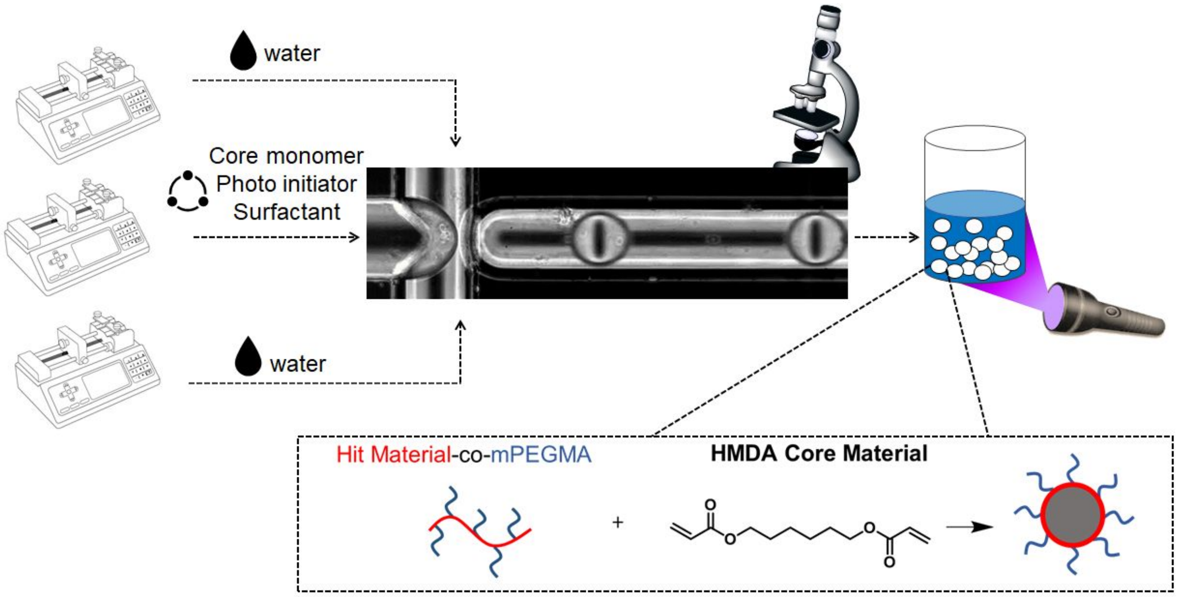

Figure S1 Schematic of the experimental microfluidics setup. The organic and aqueous phase were pumped with 3 syringe pumps and connected to the device via polytetrafluoroethylene tubes. A high-speed video camera was applied to observe the droplet formation through the flow focusing microfluidic chip. The droplets were collected in a glass vessel with water and shine by UV-Vis. At the bottom of the picture, it was included a generic schematic

of the chemistry used in this set up. The result in the application of the custom-made functionalised surfactants with an inert diacrylate monomer is the formation of cross-linked MPs (60-70 µm) with a low-density PEG-layer on the surface (around 10 nm).

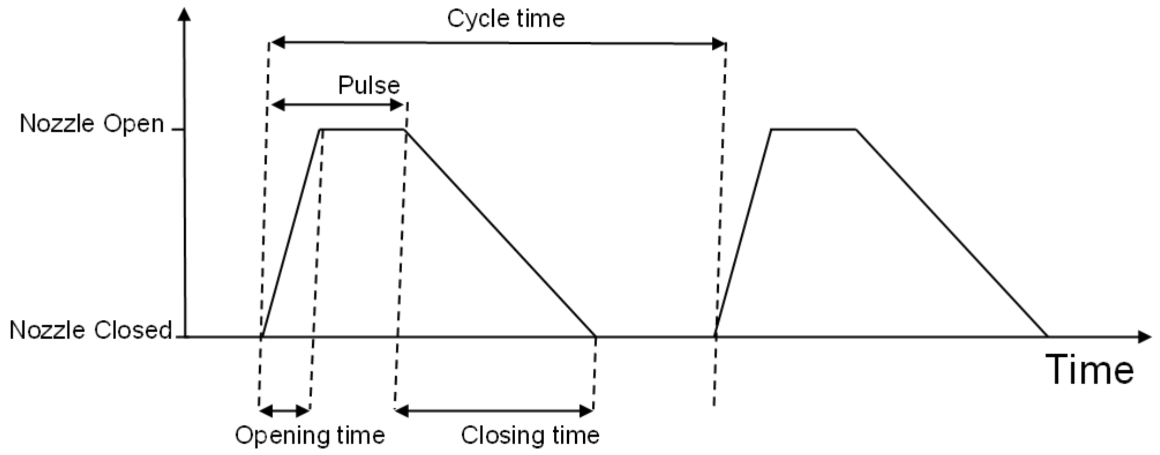

Scheme S2. A typical unipolar waveform for valve-based jetting. Based on the rheological properties of the inks, the settings were optimised to be close, pulse and cycle times of 0.6 ms, 0.5 ms, and 5 ms, respectively.

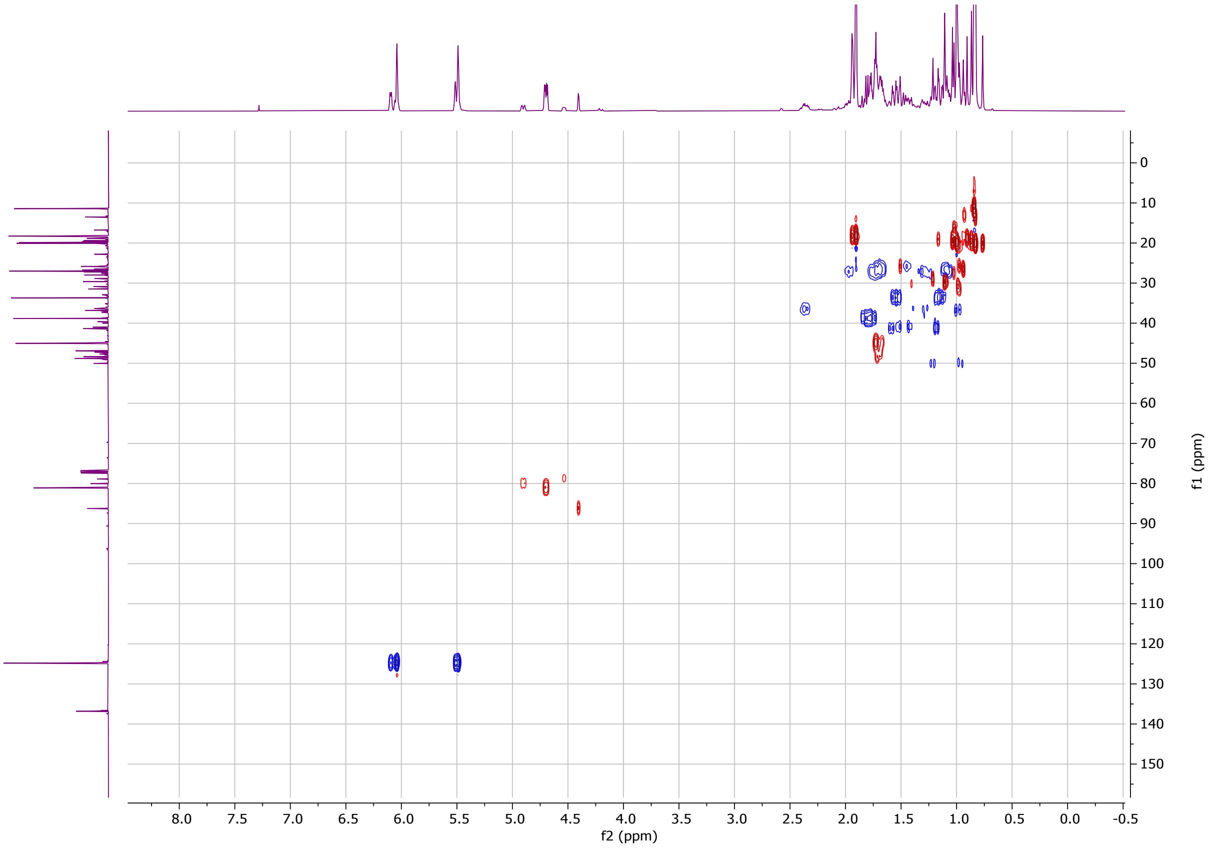

Figure S2 2D-HSQC NMR spectrum used to qualitatively evaluate the nature of product mixture obtained from the iron triflate catalysed reaction of (-)-α-pinene and methacrylic acid.

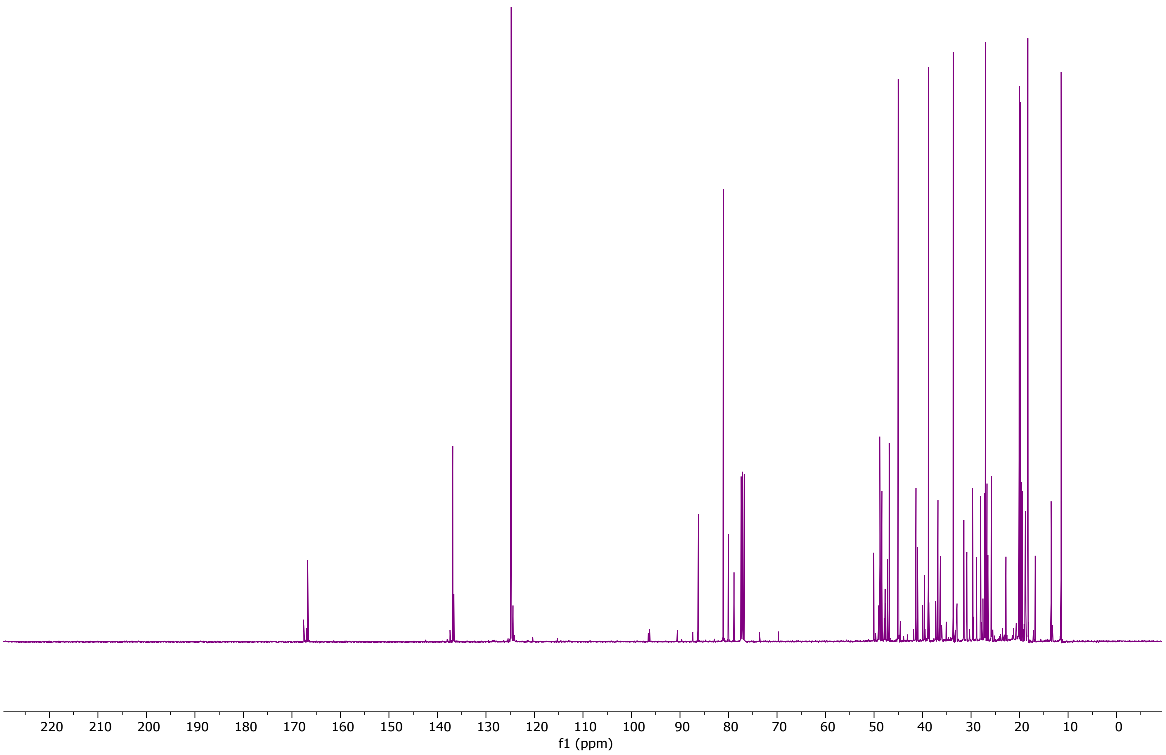

Figure S3 ^{13}C-NMR spectrum used to qualitatively evaluate the nature of product mixture obtained from the iron triflate catalysed reaction of (-)-α-pinene and methacrylic acid.

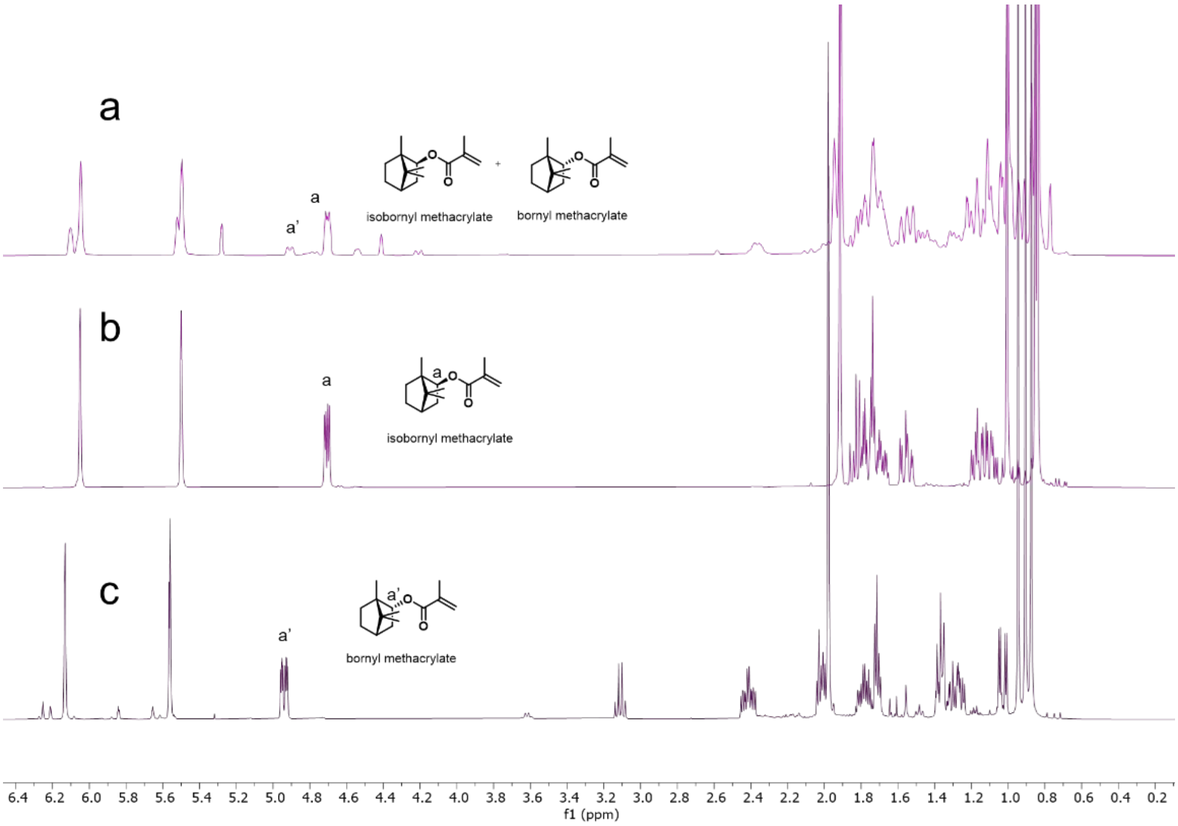

Figure S4 Comparison the ^{1}H-NMR spectra of (a) the iron triflate synthesised mixture of isobornyl and bornyl methacrylate, (b) pure isobornyl and (c) pure bornyl methacrylate monomers

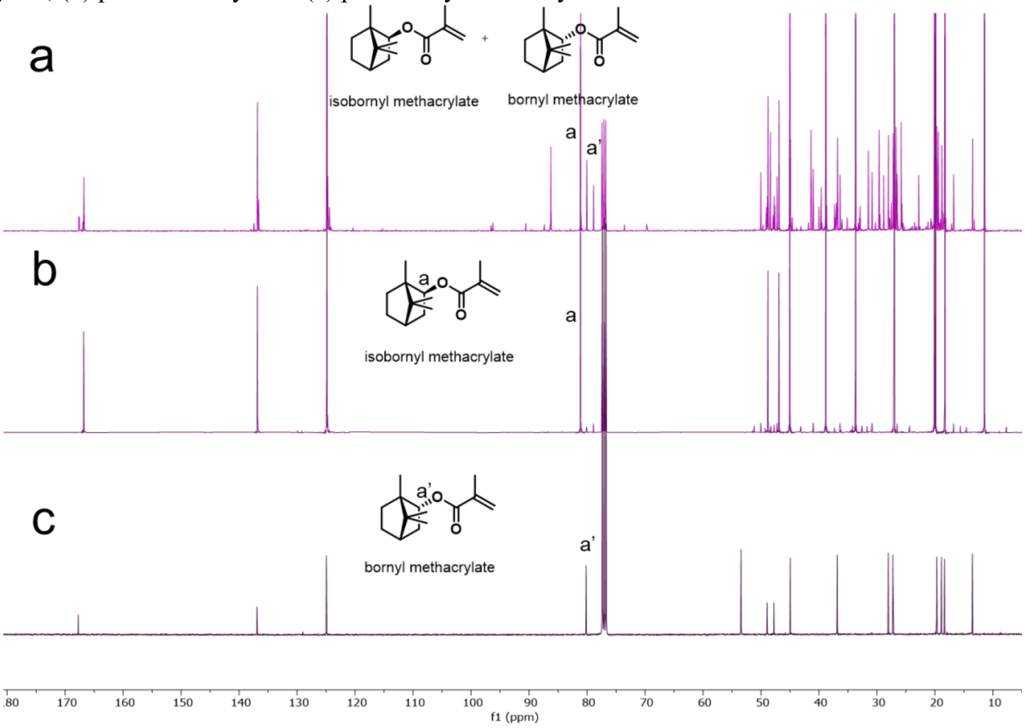

Figure S5 Comparison the ^{13}C-NMR spectra of (a) the iron triflate synthesised mixture of isobornyl and bornyl methacrylate, (b) pure isobornyl and (c) pure bornyl methacrylate monomers

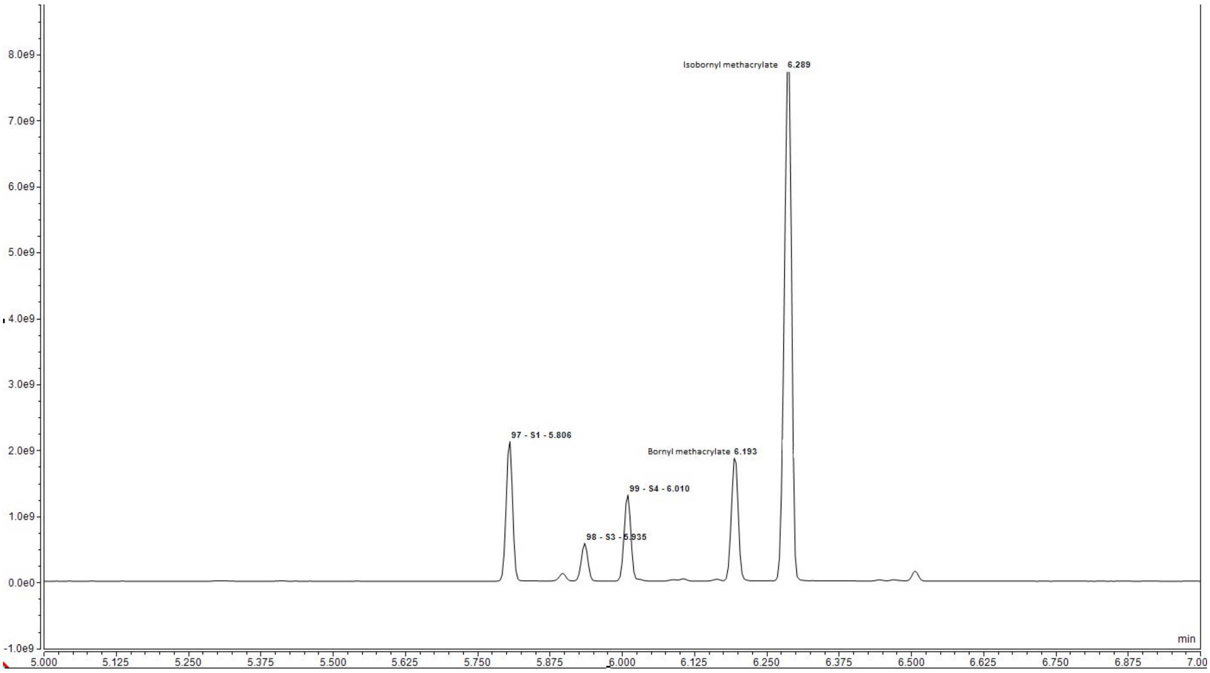

Figure S6 GC-MS analysis data collected from the reaction product of pinene and methacrylic acid.

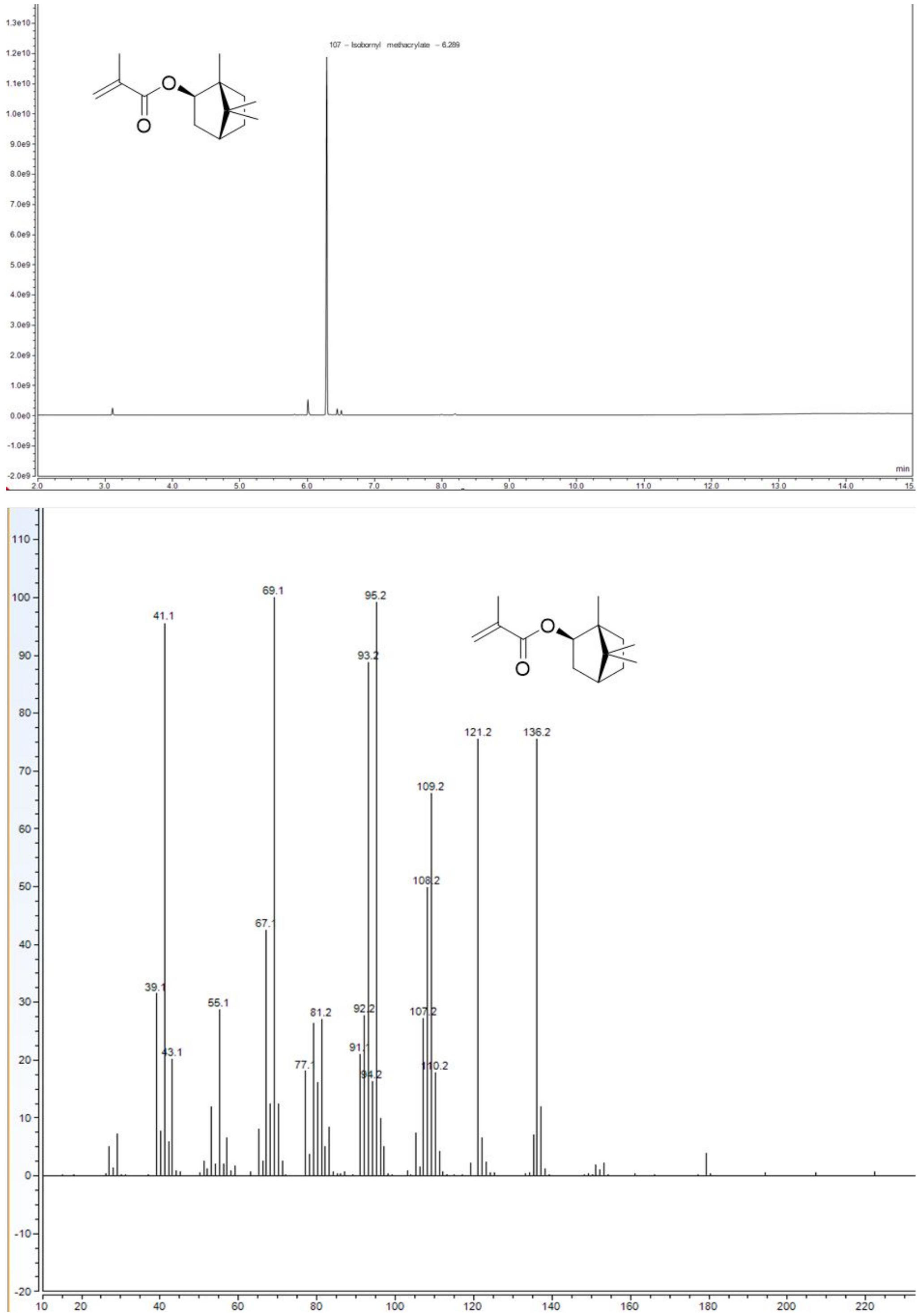

Figure S7 GC-MS analysis data collected from authentic samples of isobornyl methacrylate

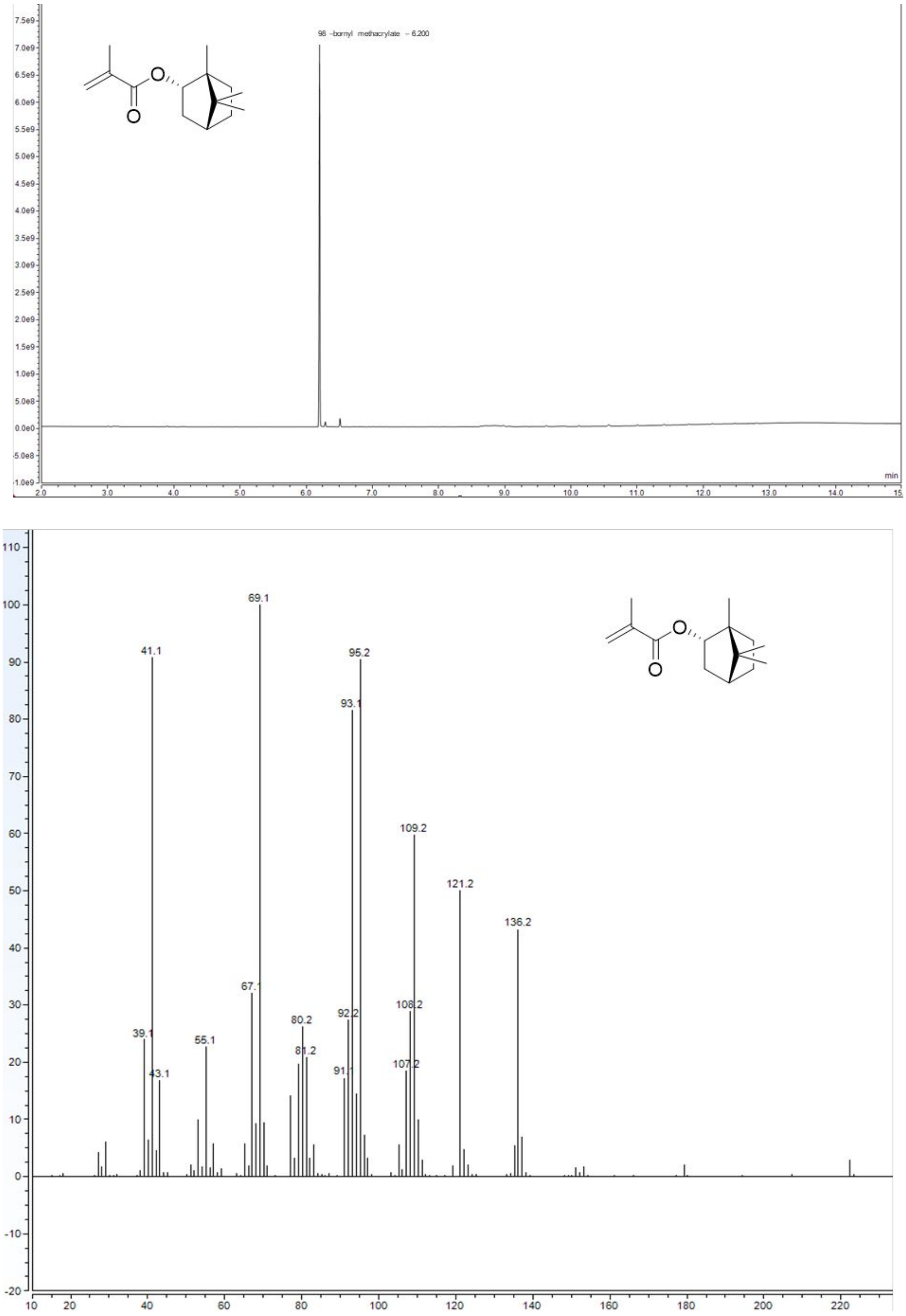

Figure S8 GC-MS analysis data collected from authentic samples of bornyl methacrylate

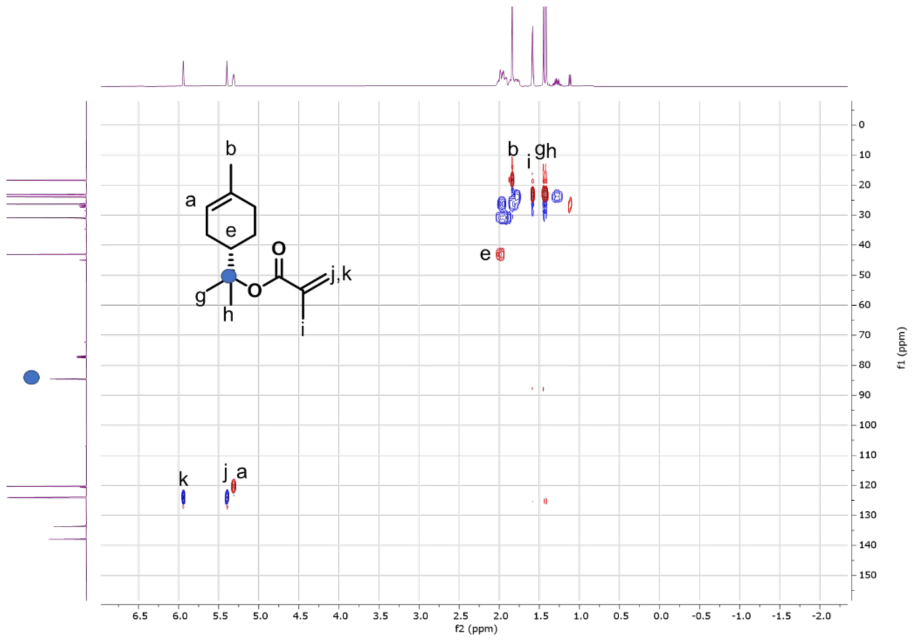

Figure S9 The HSQC spectrum which confirmed the structure of the αTMA monomer

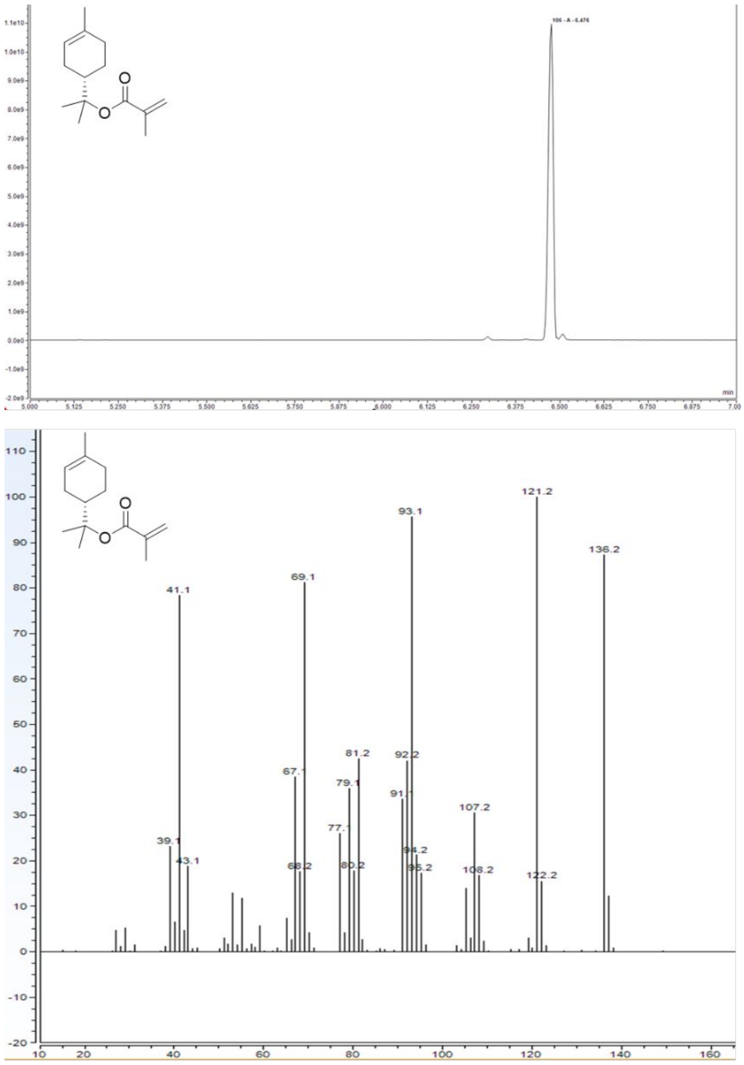

Figure S10 GC-MS analysis data collected from authentic samples of αTMA
